# Transcriptome Changes in Glioma Cells upon Infection with the Oncolytic Virus VV-GMCSF-Lact

**DOI:** 10.3390/cells12222616

**Published:** 2023-11-12

**Authors:** Dmitriy V. Semenov, Natalia S. Vasileva, Maya A. Dymova, Sergey V. Mishinov, Yulya I. Savinovskaya, Alisa B. Ageenko, Anton S. Dome, Nikita D. Zinchenko, Grigory A. Stepanov, Galina V. Kochneva, Vladimir A. Richter, Elena V. Kuligina

**Affiliations:** 1 Institute of Chemical Biology and Fundamental Medicine, Siberian Branch, Russian Academy of Sciences, Lavrentyev Avenue, 8, 630090 Novosibirsk, Russia; nataly_vas@bk.ru (N.S.V.); maya.a.rot@gmail.com (M.A.D.); yulya_savinovskaya@mail.ru (Y.I.S.); a.ageenko@g.nsu.ru (A.B.A.); domeanton@yandex.ru (A.S.D.); nikita.zinchenko.1994@mail.ru (N.D.Z.); stepanovga@niboch.nsc.ru (G.A.S.); richter@niboch.nsc.ru (V.A.R.); kuligina@niboch.nsc.ru (E.V.K.); 2 Novosibirsk Research Institute of Traumatology and Orthopedics n.a. Ya.L. Tsivyan, Department of Neurosurgery, Frunze Street, 17, 630091 Novosibirsk, Russia; smishinov@yandex.ru; 3 State Research Center of Virology and Biotechnology “Vector”, Rospotrebnadzor, 630559 Koltsovo, Russia; kochneva@vector.nsc.ru

**Keywords:** glioma, glioblastoma, patient-derived cell cultures, virotherapy, vaccinia virus, oncolytic virus, VV-GMCSF-Lact, next-generation sequencing, transcriptome, differentially expressed genes

## Abstract

Oncolytic virotherapy is a rapidly evolving approach that aims to selectively kill cancer cells. We designed a promising recombinant vaccinia virus, VV-GMCSF-Lact, for the treatment of solid tumors, including glioma. We assessed how VV-GMCSF-Lact affects human cells using immortalized and patient-derived glioma cultures and a non-malignant brain cell culture. Studying transcriptome changes in cells 12 h or 24 h after VV-GMCSF-Lact infection, we detected the common activation of histone genes. Additionally, genes associated with the interferon-gamma response, NF-kappa B signaling pathway, and inflammation mediated by chemokine and cytokine signaling pathways showed increased expression. By contrast, genes involved in cell cycle progression, including spindle organization, sister chromatid segregation, and the G2/M checkpoint, were downregulated following virus infection. The upregulation of genes responsible for Golgi vesicles, protein transport, and secretion correlated with reduced sensitivity to the cytotoxic effect of VV-GMCSF-Lact. Higher expression of genes encoding proteins, which participate in the maturation of pol II nuclear transcripts and mRNA splicing, was associated with an increased sensitivity to viral cytotoxicity. Genes whose expression correlates with the sensitivity of cells to the virus are important for increasing the effectiveness of cancer virotherapy. Overall, the results highlight molecular markers, biological pathways, and gene networks influencing the response of glioma cells to VV-GMCSF-Lact.

## 1. Introduction

Adult-type diffuse gliomas represent one of the most common types of central nervous system tumors and occur in 5–6 cases per 100,000. Currently, the first-line treatment for grade III–IV gliomas is surgical resection followed by radiotherapy and chemotherapy temozolomide, but this does not significantly increase the life expectancy of patients [1].

Therapy with oncolytic viruses is an actively developing approach to cancer treatment, allowing for the selective targeting and lysis of tumor cells [2]. In addition to direct oncolysis, virotherapy induces an antitumor immune response. Tumor-associated antigens are released during cell lysis, leading to their recognition by the immune system and the recruitment of activated immune cells [3]. At present, various oncolytic viruses are undergoing preclinical and clinical trials as therapies for glioblastoma. Moreover, the recent approval of the oncolytic herpes virus G47∆ as a pioneering glioblastoma treatment in Japan emphasizes the expediency of further research [4].

Underlying the development of oncolytic approaches, the vaccinia virus (VACV) has the principal advantage of a natural tropism for tumors of various histogenesis, as it does not require specific receptors to penetrate the cell [5]. A main strategy for enhancing the oncolytic effect of VACV involves inserting transgenes into the virus genome. The introduced sequences can encode proteins that induce apoptosis or mediate recruiting host immune cells and enhance the antitumor immune response. In particular, the selective targeting of VACV to a tumor is ensured by the optimal conditions for successful virus replication that are created by the hallmarks of malignant cell transformation (e.g., disturbances in the apoptotic pathways, cell cycle dysregulation, and immune system evasion). In addition, VACV replication and dissemination depend on the epidermal growth factor receptor (EGFR) signaling pathway, which is activated in most tumors [6].

JX-594, or Pexa-Vec, is one of the most well-known and studied recombinant VACVs used as a candidate drug for the treatment of malignant neoplasms [7]. This modified virus lacks the viral thymidine kinase (tk) gene but carries human GM-CSF and β-galactosidase genes under the control of synthetic early/late and p7.5 promoters, respectively. The inactivation of the thymidine kinase gene makes virus replication dependent on high thymidine kinase activity in the cell, a characteristic feature of tumor cells [8]. GM-CSF expressed by the virus stimulates the antitumor immune response [9]. Indications for using Pexa-Vec in clinical trials include hepatocellular carcinoma, melanoma, breast cancer, and other solid tumors. The drug has demonstrated safety with various routes of administration; however, clinical approval has not yet been obtained.

Previously, we have developed *VV-GMCSF-Lact*, a recombinant VACV derived from the parent Lister (L-IVP) strain. *VV-GMCSF-Lact* is considered a promising drug for the treatment of solid tumors, including glioblastoma [10]. In this viral construct, the viral thymidine kinase (tk) and growth factor (vgf) genes are replaced with human genes encoding GM-CSF and the apoptosis-inducing protein lactaptin, respectively [10]. Lactaptin is a proteolytic fragment of human kappa-casein. It has proapoptotic activity against tumor cells, inducing apoptosis via the mitochondrial pathway and autophagy [11]. *VV-GMCSF-Lact* is currently undergoing clinical trials as an oncolytic treatment for breast cancer, including triple-negative (ClinicalTrials.gov Identifier: NCT05376527). In addition, we have shown that *VV-GMCSF-Lact* exhibits high oncolytic activity in vitro, as well as antitumor efficacy against human glioma *in vivo*. Its ability to penetrate the blood–brain barrier has been established both in mice with tumors and in intact mice [12]. Since cells of different glioma cultures can show different sensitivity to *VV-GMCSF-Lact*, it is necessary to study the mechanisms that determine the sensitivity of glioma cells to the virus in order to develop the most effective therapeutic approaches.

Here, to describe the effect of *VV-GMCSF-Lact* on the human cell transcriptome, we used patient-derived glioma/glioblastoma cell cultures (BR1, BR3, BR4, BR5), immortalized U87 and U343 human glioblastoma cell lines, and a patient-derived non-malignant human brain cell culture (NB). By analyzing sets of differentially expressed genes (DEGs), we determined general changes in the transcriptome and identified individual clusters of genes associated with sensitivity or resistance of glioma cells to the cytotoxic activity of *VV-GMCSF-Lact*.

## 2. Materials and Methods

### 2.1. Immortalized Cell Lines

Human U87 MG and U343 MG cell lines were purchased from the Russian cell culture collection (Russian Branch of the ETCS, St. Petersburg, Russia). The cells were cultivated in Minimum Essential Medium α (MEM α; Sigma-Aldrich, MS, USA) supplemented with 10% FBS (Gibco BRL Co., Gaithersburg, MD, USA), 2 mM L-glutamine (Sigma-Aldrich, MS, USA), 250 mg/mL amphotericin B, and 100 U/mL penicillin/streptomycin (Gibco BRL Co., Gaithersburg, MD, USA) at 37 °C in a humidified atmosphere containing 5% CO_2_.

### 2.2. Patient-Derived Cell Cultures

Glioma tissue samples were obtained at the Novosibirsk Research Institute of Traumatology and Orthopedics (Novosibirsk, Russia) from patients who provided informed consent. The study was approved by the Committee on the Ethics of the Novosibirsk Research Institute of Traumatology and Orthopedics (protocol no. 050/17 68 of 11 September 2017). A sample of normal brain tissue (NB) was obtained from a patient without a malignant tumor at the time of surgery. Glioma samples (BR1, BR3, BR4, and BR5) and NB were mechanically dissociated in Iscove’s modified Dulbecco’s media (IMDM, Sigma-Aldrich, MS, USA). Then, specimens were washed with 10X excess of phosphate-buffered saline (PBS) and collected with centrifugation at 300× *g*. Specimens were seeded in 6-well plates using IMDM medium supplemented with 10% FBS, 2 mM L-glutamine, 100 U/mL penicillin, 100 µg/mL streptomycin, and 250 mg/mL amphotericin B. They were then incubated at 37 °C in a humidified atmosphere containing 5% CO_2_ for cell adhesion, and the medium was changed every three to four days. After reaching 70–80% confluence, cells were harvested using Triple-Express (GIBCO, Thermo Fisher, Waltham, NY, USA) and subcultured for further experiments. In the following steps, the cells were cultured under the same conditions.

### 2.3. Oncolytic Virus VV-GMCSF-Lact

The recombinant *VV-GMCSF-Lact* was engineered from the VACV Lister strain (L-IVP) and contained deletions of the viral thymidine kinase (tk) and virus growth factor (vgf) gene fragments. In the corresponding regions, we inserted the gene of human GM-CSF (CSF2) and the gene of the proapoptotic fragment of human kappa-casein (CSN2) or lactaptin [10]. *VV-GMCSF-Lact* was produced in African green monkey kidney cells 4647 and purified as described in [13]. The viral titer was determined using the plaque-forming assay and expressed as a number of plaque-forming units per volume (i.e., PFU/mL) [13].

### 2.4. Flow Cytometry

Cells of immortalized (U87 MG and U343 MG) and patient-derived (BR1, BR3, BR4, BR5, NB) cultures grown in 6-well plates were collected and incubated with phycoerythrin (PE)-conjugated mouse anti-human CD133 mAbs (Miltenyi Biotec, Bergisch Gladbach, Germany), fluorescein isothiocyanate (FITC)-conjugated rat anti-human CD44 antibody (Invitrogen, CA, USA), Alexa Fluor 488-conjugated mouse anti-human/mouse CD15 antibody (R&D Systems, Minneapolis, MN, USA), and PE-conjugated mouse anti-human CD171 antibody (Sony Biotechnology, San Jose, CA, USA) in PBS supplemented with 0.5% fetal bovine serum and 2 mM EDTA for 30 min on ice. The assay was run on a BD FACSCantoII flow cytometer (BD Biosciences, Franklin Lakes, NJ, USA), and the data were analyzed using the BD FACSDiva Software (BD Biosciences, Franklin Lakes, NJ, USA).

### 2.5. XCelligence Assay

The cell proliferation and adhesion kinetics were determined using RTCA technology (ACEA Biosciences, San Diego, CA USA). Cells of immortalized (U87 and U343) and patient-derived (BR1, BR3, BR4, BR5, NB) cultures were seeded (30,000 cells/well) in an 8-well E-plate in three technical replicates. The plate was incubated in a humidified atmosphere containing 5% CO_2_ at 37 °C for 72 h, and the cell index for each culture was automatically tracked during this period.

### 2.6. Apoptosis Detection

Cells of immortalized (U87 and U343) and patient-derived (BR1, BR3, BR4, BR5, NB) cultures were treated with *VV-GMCSF-Lact* at a multiplicity of infection (MOI) of 1 PFU per cell. Following 24 h incubation with the virus, cells were harvested using Triple-Express (GIBCO, Thermo Fisher, Waltham, NY, USA) and stained with annexin V-FITC and PI using the BD Pharmingen Apoptosis Detection Kit (BD Bioscience, Franklin Lakes, NJ, USA) according to the manufacturer’s protocol. Cells without any virus exposure were used as controls. The analyses were performed using the BD FACSCantoII flow cytometer (BD Biosciences, Franklin Lakes, NJ, USA), and the data were analyzed using the BD FACSDiva Software (BD Biosciences, Franklin Lakes, NJ, USA).

### 2.7. Determination of the Cytotoxic Dose (CD_50_) of VV-GMCSF-Lact for Glioma and Normal Brain Cell Cultures

To study the cytotoxic activity of *VV-GMCSF-Lact*, cells of immortalized (U87 MG and U343 MG) and patient-derived (BR1, BR3, BR4, BR5, NB) cultures were plated into 96-well plates, 3000 cells per well. Then, cells were treated with *VV-GMCSF-Lact* at an MOI ranging from 10 to 0.0012 PFU per cell using a two-step dilution factor. After 72 h incubation at 37 °C in a humidified atmosphere containing 5% CO_2_, cell viability was evaluated using the Deep Blue Cell Viability™ Kit (Biolegend, CA, USA). Cell viability was determined relative to the viability of the control cells (100% ± standard deviation in three independent experiments). The cytotoxic dose (CD_50_, when 50% of cells die) was calculated using the Compusyn software [14].

### 2.8. Treatment with VV-GMCSF-Lact for Next-Generation RNA Sequencing

To obtain RNA samples for the next-generation RNA sequencing (NGS), the cells of immortalized (U87 and U343) and patient-derived (BR1, BR3, BR4, BR5, NB1) cultures were first plated into 6-well plates, with 1 million cells per well. Then, cells were treated with 1 PFU per cell of *VV-GMCSF-Lact*. After 12 h and 24 h incubation at 37 °C in a humidified atmosphere containing 5% CO_2_, cells were harvested using Triple-Express (GIBCO, Thermo Fisher, Waltham, NY, USA). Cells incubated in the same conditions but without exposure to the virus were used as the control.

### 2.9. RNA Isolation

To isolate total RNA, an RNA extraction kit (LRU-100-50, Biolabmix, Russia) was used according to the manufacturer’s protocol. RNA concentration was assessed using the Qubit 2 fluorometer (Thermo Fisher Scientific, USA) and the Qubit RNA HS Assay Kit (Thermo Fisher Scientific, USA). The quality of total RNA, expressed as the RNA integrity number (RIN), was determined using Bioanalyzer 2100 (Agilent, USA) with the Agilent RNA Pico 6000 Kit (Agilent, USA). A threshold RIN value greater than 8.0 was taken as the cutoff point for moving to the library preparation stage.

### 2.10. RNA Sequencing

Illumina cDNA libraries were produced according to a standard protocol using the NEBNext Ultra II Targeted RNA Library Preparation Kit (New England Biolabs, UK) and the NEBNext mRNA Magnetic Isolation Module (New England Biolabs, UK), as well as mass parallel sequencing on the NextSeq Illumina platform 1500 at the Institute of Fundamental Medicine and Biology of the Kazan Federal University (Kazan, Russia). For mRNA isolation, fragmentation, and priming, 1 μg of total RNA was used. The NextSeq 500/550 High Output v2.5 Kit (Illumina, USA) creating 100-nucleotide single-end reads was used. Fragment size distribution in the prepared sequencing libraries was analyzed using the Bioanalyzer 2100 instrument (Agilent, USA) with the Agilent High Sensitivity DNA Kit (Agilent, USA) and quantified using the Qubit 2.0 Fluorometer (Invitrogen, USA) with the Qubit dsDNA HS Assay Kit (Thermo Fisher Scientific, USA). Fragment sizes varied from 250 to 700 bp with a clear peak at 300 bp.

### 2.11. Transcriptome Analysis

Raw sequencing reads (76-nucleotide single-end reads) were subjected to Illumina adapter removal using Trimmomatic [15]. The trimmed sequencing reads were filtered with Bowtie2 [16] using a reference containing sequences of human rRNAs (RefSeq); tRNAs; snRNA; SINE-, LINE-, and DNA-repeat consensus sequences (RepBase [17]); low-complexity simple repeats; and mitochondrial DNA (NC_012920.1). The filtered reads were mapped to the human genome (GRCh38/hg38) with the addition of the *VV-GMCSF-Lact* genome as a single chromosome. The CSF3 and CSN3 (corresponding to lactaptin) gene sequences were excluded from the human genome by masking the corresponding regions with N. Filtered reads were mapped with STAR 2.7.1a [18] using the RefGene human genome annotation (https://hgdownload.soe.ucsc.edu/goldenPath/hg38/bigZips/genes/, accessed on 3 March 2022) modified with the *VV-GMCSF-Lact* genome annotation. To construct the *VV-GMCSF-Lact* genome annotation, we used the annotation of the parental VACV strain L-IVP (GenBank accession KP233807) and experimental data from [10]. Aligned reads were quantified using QoRTs v1.3.6 [19]. Differential gene expression analysis was performed with DESeq2 1.36.0 [20], R version 4.1.3, and Bioconductor 3.14. Following the differential gene expression analysis, lists of upregulated and downregulated genes were analyzed with Enrichr using the R interface [21].

## 3. Results

### 3.1. Cell Cultures and General NGS RNA-Seq Data

#### 3.1.1. Cell Cultures

Immortalized cell lines are commonly used for studying the emergence and growth of malignant tumors. However, these models lose their ability to reflect the inherent characteristics of the primary tumor due to long-term cultivation, whereas cultures obtained from patients retain cellular heterogeneity and the original biological characteristics of the tumor [22,23]. Thus, patient-derived cultures enable assessing the effect of therapeutic agents on tumor cells more accurately and help predict their clinical efficacy.

**Figure 1:**
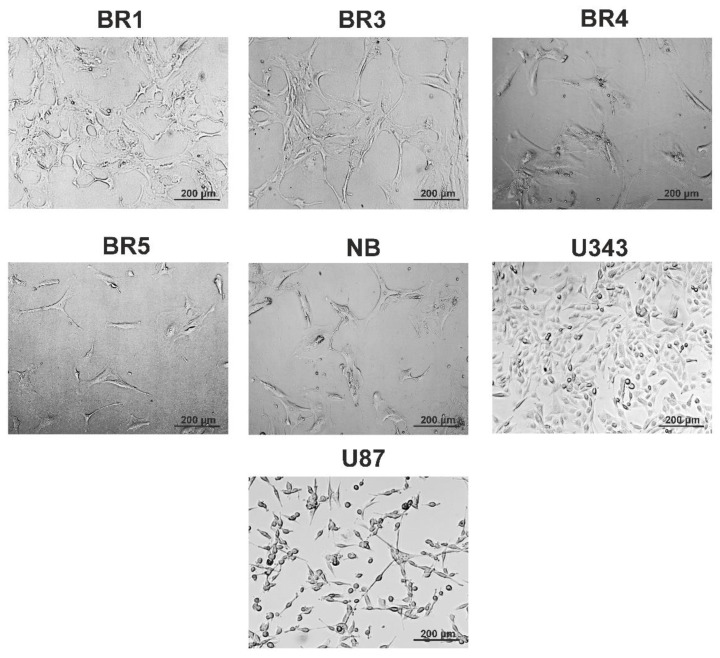
Representative images of immortalized human glioblastoma lines (U343, U87), NB cells, and patient-derived glioma cell cultures (BR1, BR3, BR4, BR5).

In this work, we used two immortalized cell lines—U87 MG (U87) and U343 MG (U343)—as established glioma models. Additionally, patient-derived glioma cultures BR1, BR3, BR4, and BR5 were obtained from patient tumors. NB was a primary cell culture derived from non-tumor human brain tissue obtained during surgery. These cells exhibited typical glial morphology (Figure 1).

The cell cultures were evaluated for the presence of cancer stem cell markers (CD44, CD133, CD15, and CD171) and cell proliferation rate (Table 1). According to our data, the cell growth rate and the levels of stem cell markers are independent of each other or the primary tumor grade.

Levels of cancer stem cell markers are different in the cell cultures studied. The CD133+/CD44+ population, which seems to be the most common for cancer stem cell identification, is the highest in the BR1 culture (14.2%). BR4 is the only cell culture that is characterized by the presence of all cancer stem cell markers (9.92% CD133+, 78.9% CD44+, 9.56% CD133+/CD44+, 20.4% CD15+, 1.95% CD171+; Table 1). It is worth noting that the NB culture contains abundant CD44+ (99.7%) and CD171+ (44.1%) cell populations. These markers are usually discussed in the context of their significance in malignant neoplasms [24,25,26,27]. However, CD44 and CD171 are both normally expressed in the nervous system because these molecules play an important role in its functioning [28,29].

According to the data from the iCELLigence real-time cell analysis, the most slowly proliferating cells are NB (Cell Index (CI) is 1.07) and BR4 (CI = 0.94), while BR1 cells have a higher proliferation rate (CI = 11.12). Thus, the cell growth rate and the level of stemness markers depend neither on each other nor on the grade of the primary tumor.

Next, we evaluated the cytotoxic activity of *VV-GMCSF-Lact* (Table 1). Cells of various glioma cultures and NB cells show different sensitivity to the virus. Sensitivity decreases in the series BR1 > BR3 > U343 > U87 > BR5 > BR4 >> NB, and benign NB cells are characterized by relatively high resistance to the virus (5.8 PFU/cell versus 6.3E-3 PFU/cell for BR1). This implies that glioma cells have different sensitivity to *VV-GMCSF-Lact*, which does not depend on the grade of malignancy or the presence of cancer stem cell markers (Table 1).

To analyze the apoptotic processes in glioma and NB cells during viral infection, cells were treated with *VV-GMCSF-Lact* (MOI of 1 PFU per cell) for 24 h. Following staining with annexin V and propidium iodide, the cells were analyzed with flow cytometry. The results suggest that upon infection with the virus, the cells died mainly via apoptosis. After 24 h incubation, we observed a significant population of apoptotic AnnV+/PI- cells in most of the studied cultures.

**Figure 2:**
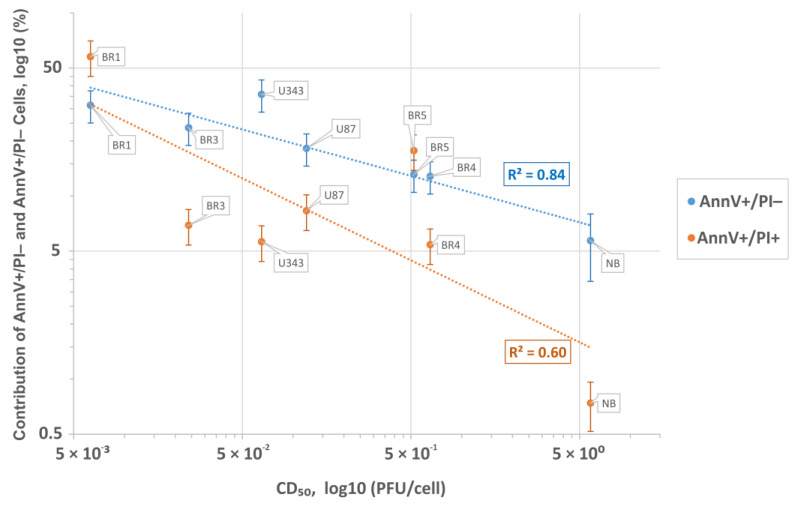
Inverse correlation between the sensitivity of glioma and NB cells to the cytotoxic dose of *VV-GMCSF-Lact* (CD_50_). The percentage of apoptotic (AnnV+/PI-) and necrotic (AnnV+/PI+) cells, 12 h after the infection with 1 PFU/cell of the virus. The mean values from three independent measurements and the corresponding standard deviations are presented.

The percentage of both Ann+/PI- and Ann+/PI+ cells were found to negatively correlate with the virus cytotoxicity CD_50_ (Pearson R^2^ = 0.84 and R^2^ = 0.60, respectively, Figure 2). Thus, the proapoptotic response of glioma and NB cells correlates well with their sensitivity to the cytotoxic effect of *VV-GMCSF-Lact*, as determined by the cytotoxic dose (CD_50_).

#### 3.1.2. RNA-Seq Data

To analyze processes occurring in human glioma and NB cells upon infection with *VV-GMCSF-Lact*, we used NGS-transcriptome analysis on the Illumina 1500 NGS platform. High-throughput sequencing data for the polyA-enriched RNA fractions was obtained, containing from ~20 to 47 million sequencing reads for each cell culture 0, 12, and 24 h after the infection (Table S1).

**Figure 3:**
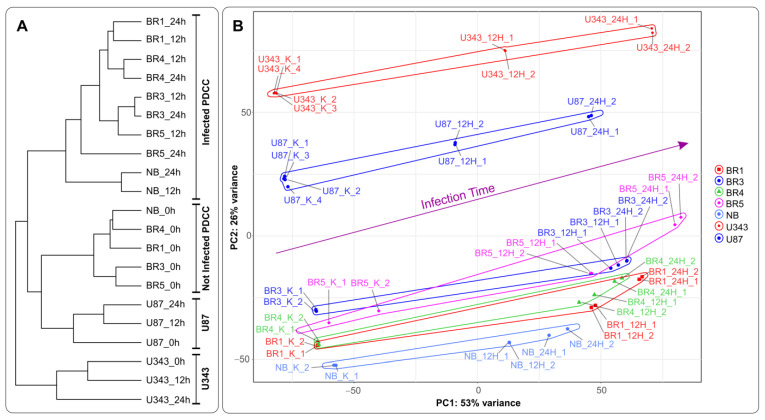
(**A**) A tree of Euclidean distances of variance stabilizing transformed (VST) RNA expression data of immortalized glioblastoma, patient-derived glioma cell cultures, and NB cells. The complete agglomeration method was used for clustering. (**B**) Principal component analysis of DESeq2 normalized VST-transformed RNA expression data. Sample-specific PC1:PC2 points are annotated with cell-defined envelopes. The deep purple arrow shows the common trend of PC1:PC2 transition from noninfected (0 h) to the 12 h and 24 h *VV-GMCSF-Lact*-infected state. Patient-derived cell cultures (PDCCs).

In terms of hierarchical clustering (HC), transcriptomic changes upon *VV-GMCSF-Lact* infection generally show that immortalized cell lines, U343 and U87, form two independent branches on the tree for both infected and noninfected cells. In contrast, RNA patterns of patient-derived glioma cultures (BR1, BR3, BR4, BR5) and NB cells can be clearly divided into two groups, corresponding to the noninfected and the *VV-GMCSF-Lact*-infected HC branches (Figure 3A).

In agreement with HC, the principal component analysis (PCA) shows that formalized RNA expression data for immortalized cell lines (U343, U87) form two non-overlapping regions in terms of PC1:PC2, with a common trend of changes during infection (Figure 3B). Patient-derived glioma cell cultures (BR1, −3, −4, and −5) form a cloud of partially overlapping PC1:PC2 dots distinguishable from NB cells. However, there is a common trend of changes in the PC1:PC2 coordinates for all analyzed gliomas and NB cells when infected with *VV-GMCSF-Lact* (Figure 3B). Thus, the virus infection promotes common transcriptional changes in all analyzed gliomas and NB cells, sharing gene patterns, transcription factors (TFs), and/or gene functions.

### 3.2. General Changes in Individual Transcript Levels, Transcription Factor Activity, Biological Processes, and Pathways Affected by VV-GMCSF-Lact Infection

To describe the overall transcriptome changes in human glioma and NB cells during infection with *VV-GMCSF-Lact*, we employed a cell-specific approach using DESeq2. It allowed us to compare transcriptome changes in each cell culture at 12 h and 24 h time points after infection. In cell-specific DESeq2 comparisons, we determined the number of differentially expressed transcripts (or differentially expressed genes, DEGs) for each cell culture. The highest variation in RNA patterns at 24 h was observed in the BR1 cell culture (6587 DEGs), whereas U87 and NB cells, which are relatively resistant to *VV-GMCSF-Lact*, demonstrated the lowest variations (1384 and 1563 DEGs, respectively) (Table S2).

**Figure 4:**
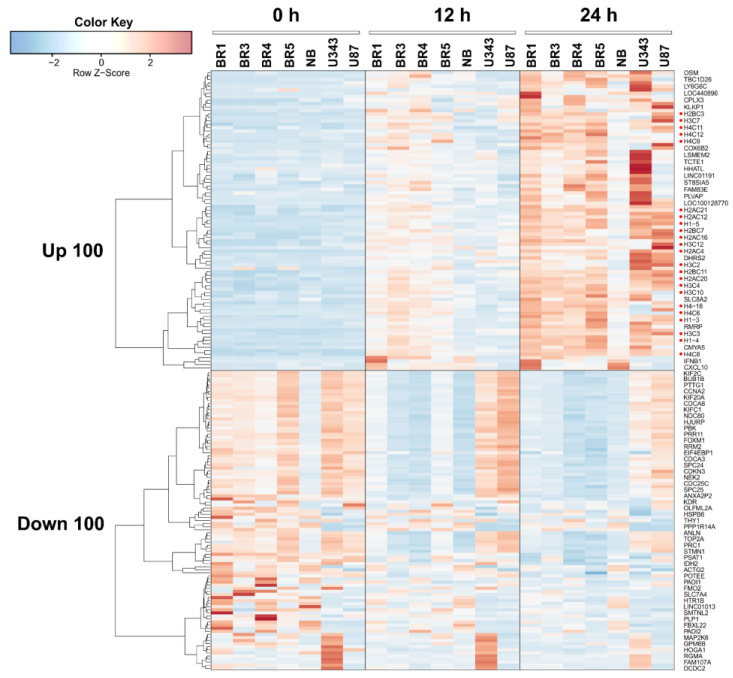
Heatmap of the 100 most variable upregulated and downregulated differentially expressed genes in glioma and NB cell cultures 12 h and 24 h after *VV-GMCSF-Lact* infection. Histone gene transcripts are marked with red dots.

We constructed a heat map showing the most variable upregulated and downregulated DEGs in glioma and NB cell lines 12 and 24 h after *VV-GMCSF-Lact* infection; histone gene transcripts are marked with red dots (Figure 4). When compared to other cell culture parameters (Table S2 and Table 1), the number of DEGs was found to correlate well with the relative contribution of viral transcripts (R^2^ = 0.72, Figure S1). At the same time, we found no significant correlation between the number of DEGs and CD_50_ or other cell culture parameters presented in Table 1 (Pearson R^2^ < 0.2, data not illustrated).

Thus, the cytotoxic and proapoptotic effects of *VV-GMCSF-Lact* infection are not directly associated with the overall level of viral gene expression; rather, they are determined by the activation of specific host genes and/or viral genes. Yet, it is of particular interest to describe the processes triggered by the oncolytic virus that are common across different cell cultures. Therefore, here, we provide a brief description of the common genes and general processes that are activated or suppressed during *VV-GMCSF-Lact* infection of human glioma and NB cell cultures.

It is known that viral infection is accompanied by large-scale changes in fundamental cellular processes. Different cells in a culture, organ, or tissue react differently to the virus, activating the innate and adaptive immune response. The response to the virus infection can lead to increased resistance, or the virus can enter the cell and create an environment suitable for self-replication, resulting in secondary infection of neighboring and distant cells [30].

To identify transcriptomic changes common to glioma and NB cells upon infection with *VV-GMCSF-Lact*, we compiled lists of upregulated and downregulated transcripts in different cell cultures. By 12 h of infection, the vast majority of differentially expressed transcripts were culture-specific: 65.89% and 62.92% of unique upregulated and downregulated DEGs, respectively (Table S3). Only the changes in the expression levels of five histone genes and the MARCKS gene could be considered common indicators of 12 h infection in all analyzed cell cultures (Table 2 and Table S3). However, by 24 h of infection, only 38–39% of the DEGs remained unique to a particular cell culture. The levels of 85 mRNAs increased in all cell cultures, while the levels of 54 mRNAs were reduced (Table 2 and Table S3). It should be noted that histone gene transcripts constituted a significant, almost definitive group among all common activated genes, at both 12 h and 24 h of infection (Table 2, Figure 4, discussed below).

Considering that changes in individual RNA levels only partially reflect the complex and interconnected processes of the viral infection response, we analyzed common characteristics of the up/downregulated transcript sets using the Enrichr platform [21]. According to the Enrichr “ENCODE and ChEA Consensus TFs from ChIP-X” library, IRF (IRF1, IRF8), REST, FOSL2, SRF, and RELA (member of NF-kB family) are outlined as common regulators of activated human host genes in the process of *VV-GMCSF-Lact* infection, whereas NFY (NFYA and NFYB) and E2F (E2F1, E2F4 and E2F6) transcription factor control sets of human DEGs are suppressed following infection with the virus (Table S4).

To describe common text annotations for up- or downregulated genes, a set of Enrichr libraries was selected, including GO, KEGG, MSigDB Hallmark, and Panther (Table S5). Common annotations of upregulated transcript clusters include inflammatory and antiviral response pathways, such as cytokine-mediated signaling pathway (GO:0019221); defense response to virus (GO:0051607); chemokine and cytokine activities (GO:0008009, GO:0005125, respectively); NF-kappa B signaling pathway; inflammatory response; and others. Clusters of upregulated histone transcripts form separate groups in Enrichr terms: nucleosome organization (GO:0034728), DNA binding (GO:0003677), and viral carcinogenesis. In addition, annotations of downregulated transcript clusters include cell cycle-related nuclear processes: mitotic spindle organization (GO:0007052), microtubule cytoskeleton organization involved in mitosis (GO:1902850), G2-M checkpoint, cytoskeletal regulation via Rho GTPase, and others (Table S5).

**Figure 5:**
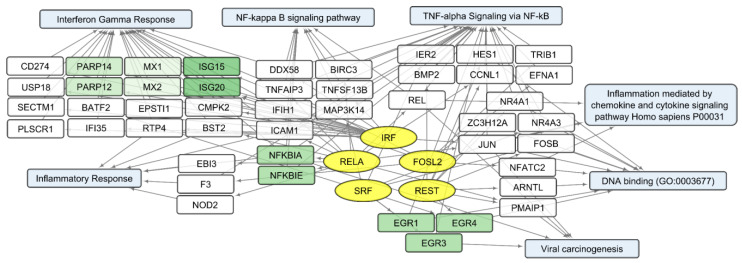
Scheme of the relationship between transcripts, transcription factors, and processes activated in glioma and NB cells upon infection with *VV-GMCSF-Lact*. Activated transcription factors IRF, RELA, FOSL2, SRF, and REST are shown in yellow ovals; selected activated genes—in white and green rectangles; and signaling pathways, biological processes, and gene annotations—in blue rectangles. Groups of genes with similar functions are drawn together using shades of green. Based on the analysis of the top 300 activated genes using Enrichr libraries: “ENCODE and ChEA Consensus TFs from ChIP-X”; “MSigDB Hallmark 2020”; “GO Biologic Process 2021”; “Panther 2016”; and “KEGG 2021 Human”.

**Figure 6:**
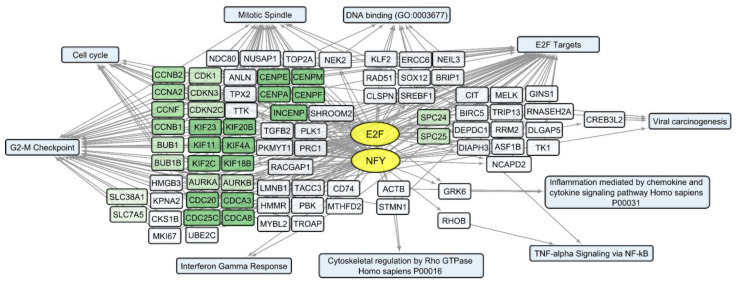
Scheme of the relationship between transcripts, transcription factors, and processes commonly suppressed in glioma and NB cells upon infection with *VV-GMCSF-Lact*. The suppressed transcription factors of the E2F family (E2F1, E2F4, and E2F6) and NFY family (NFYA and NFYB) are shown in yellow ovals; selected activated genes—in white and green rectangles; and signaling pathways, biological processes, and other gene annotations—in blue rectangles. Groups of genes with similar functions are drawn together using shades of green. Based on the analysis of the top 300 downregulated genes using Enrichr libraries: “ENCODE and ChEA Consensus TFs from ChIP-X”; “MSigDB Hallmark 2020”; “GO Biologic Process 2021”; “Panther 2016”; and “KEGG 2021 Human”.

We compared sets of DEGs regulated by selected transcription factors (Table S4) and grouped by common text annotations (Table S5). This enabled us to construct generalized schemes of activated (Figure 5) or suppressed (Figure 6) processes that are common for gliomas and NB cells upon infection with *VV-GMCSF-Lact*.

Within the sets of upregulated DEGs common to glioma and NB cells, separate groups of genes can be further distinguished. Their products are involved in the antiviral, proinflammatory response (Figure 5). These are NFKBIA and NFKBIE, feedback inhibitors of the NF-kappa-B/REL transcription factor family [31]. Members of the Early Growth Response family of transcription regulators (EGR1, 3, and 4) play an important role in the regulation of cellular responses to growth factors, DNA damage, and viral infection. The induction of EGR1 following viral infection stimulates multiple inflammatory factors (EGR2 and EGR4) and mediates host cell response to viruses [32]. Products of ISG15 and ISG20 are functionally different proteins—a ubiquitin-like modifier and an exonuclease, respectively, although both ISG15 and ISG20 belong to interferon-stimulated genes and are involved in the innate immune response to viral infection [33].

Among the human genes, whose expression is commonly suppressed in glioma and NB cells upon infection with *VV-GMCSF-Lact*, several functionally related gene clusters can be distinguished (Figure 6). These are groups of genes encoding cyclins (CCNB1, CCNB2); cyclin-dependent kinases (CDK1, CDKN3, and CDKN2C); the Aurora subfamily of cell cycle-regulated protein kinases (AURKA and AURKB); proteins associated with the cycle of cell division (CDC20, CDCA8); motor proteins of the kinesin family required for mitosis (KIF11, KIF23); and genes of centromere proteins (CENPA, CENPF).

Taken together, our data indicate that infection with *VV-GMCSF-Lact* commonly induces the expression of genes encoding elements of the innate antiviral immune response in both glioma and NB cells while suppressing various genes involved in cell division and cell cycle regulation.

### 3.3. VV-GMCSF-Lact Transcripts in Infected Glioma and NB Cell Cultures

Early VACV gene expression begins shortly after infection and persists for 2–3 h. Early genes encode the proteins of virus uncoating, transcription regulation for the subsequent phase of intermediate gene expression, and various proteins needed for the replication of the viral genome [34]. The transcription of late genes, which encode late proteins that are necessary for the assembly of new virions, starts at ~6 h post-infection [35].

In this work, we performed an NGS-transcriptomic analysis of human brain cells 12 h and 24 h after infection with the *VV-GMCSF-Lact* virus (1 PFU/cell), so as to reveal “quasi-stationary” virus transcripts detected from the onset of infection to the death of the host cell. To describe major *VV-GMCSF-Lact* genes that are commonly expressed in glioma and NB cell cultures, we ranked viral transcripts by their relative contribution in each cell culture (12 h and 24 h time points combined). We compiled a list of the top 25 genes with generally high expression by averaging and sorting the ranks assigned to individual transcripts (Table 3).

**Figure 7:**
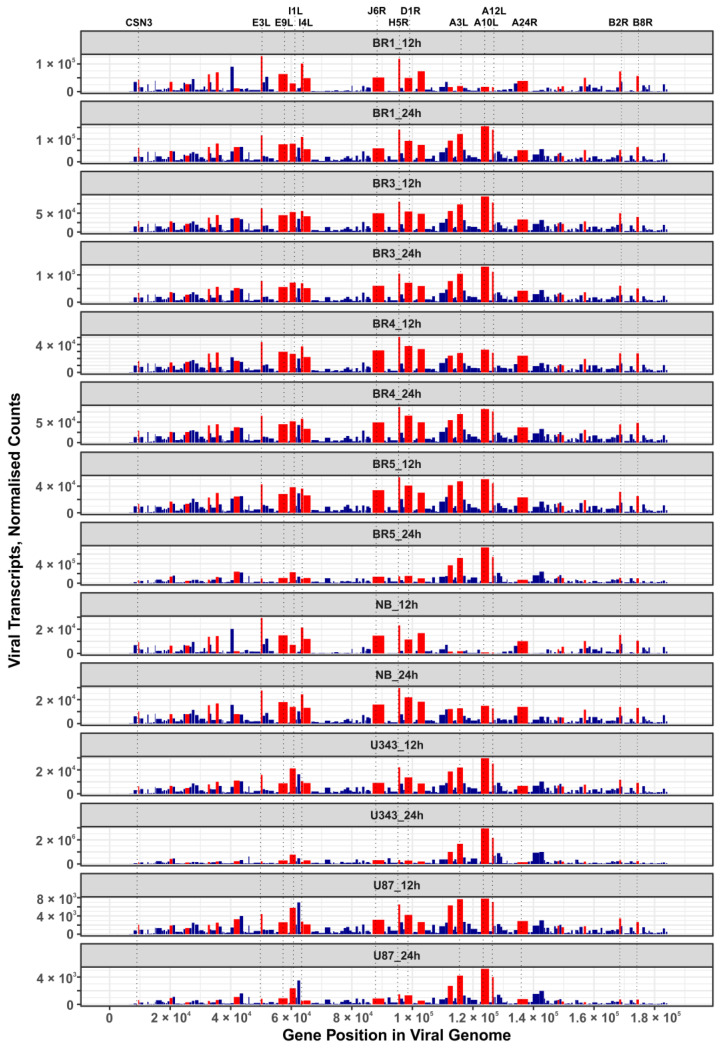
Expression maps of *VV-GMCSF-Lact* genes in glioma and NB cells. Bar plots display normalized DESeq2 RNA counts of *VV-GMCSF-Lact* transcripts (y-axis) across the length of the viral genome (x-axis). The width of the bars corresponds to the length of the gene in the virus genome. Red bars highlight the top 25 viral genes whose transcription is commonly increased in cells after 12 h and 24 h of infection (Table 3). The vertical dotted lines indicate the positions of the selected viral genes in the *VV-GMCSF-Lact* genome.

The list of VV-GMCSF-Lact transcripts expressed in both glioma cell cultures and NB cells includes products of genes that are characteristic of the VAVC “early”, “intermediate”, and “late” phases. Among the 25 commonly expressed viral genes, there are those encoding structural proteins, DNA polymerase components, host defense inhibitors, and polypeptides with other functions (Table 3). Additionally, the distribution of these 25 genes in the VV-GMCSF-Lact genome does not show any significant shifts to the 5′- or 3′-ends of viral DNA (Figure 7). Thus, VV-GMCSF-Lact genes that are commonly expressed in infected human glioma and NB cells should be described within the specific context of the host cell transcriptome, rather than solely based on their location in the virus DNA or their annotated functions (see below for further discussion).

It should also be noted that the CSN3 gene transcript encoded by *VV-GMCSF-Lact* (a fragment of the human CSN3 gene lactaptin, which we inserted into the parental VACV genome), was found among the 25 most commonly expressed viral transcripts (Table 3 and Figure 7).

### 3.4. Human Transcripts That Correlate with the VV-GMCSF-Lact RNA in Glioma and NB Cells

Apart from identifying common DEGs, another approach can help describe how cells respond to viral infection. It is based on the analysis of human transcripts whose levels correlate directly or inversely with the total level of viral transcripts. Having RNA sequencing data for the transcriptomes of seven cell cultures combined with the viral transcriptome data at each time point (Table 2), we performed a correlation analysis to identify sets of human genome transcripts whose levels correlate with the total *VV-GMCSF-Lact* RNA in infected cells. Two sets of human genome transcripts were selected: 300 transcripts showing the best positive correlation with the number of virus transcripts and 300 transcripts with the best negative correlation (representative plots are shown in Figure S3).

The set of 300 human transcripts showing the strongest positive correlation with total *VV-GMCSF-Lact* RNA levels is enriched in transcripts regulated by such transcription factors as ATF2, BRCA1, CREB1, TAF1, and YY1 (Enrichr terms form “ENCODE and ChEA Consensus TFs from ChIP-X” library, Table S6). The set of 300 negatively correlating transcripts is dominated by gene products regulated by the MYC-MAX family and the NFY family, as well as by the same transcription factors as in the case of positive correlation: ATF2, CREB1, TAF1, and YY1 (Table S6).

**Figure 8:**
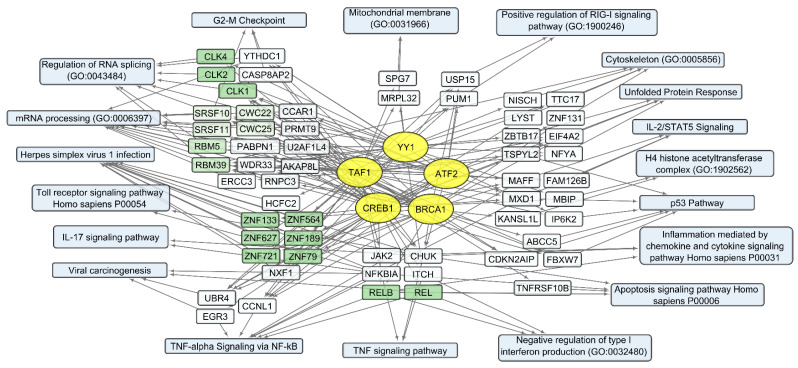
Human transcripts that positively correlate with the total *VV-GMCSF-Lact* RNA in glioma and NB cells. The scheme illustrates the relationships between the activity of transcription factors YY1, ATF2, BRCA1, CREB1, and TAF1 (yellow ovals) and transcripts that positively correlate with total *VV-GMCSF-Lact* RNA (white and green rectangles). The associated signaling pathways, biological processes, and other gene annotations are shown in blue rectangles. Groups of genes with similar functions are drawn together using shades of green based on the analysis of gene sets using the Enrichr libraries “ENCODE and ChEA Consensus TFs from ChIP-X”; “MSigDB Hallmark 2020”; “GO Biologic Process 2021”; “KEGG 2021 Human”; and “Panther 2016”.

**Figure 9:**
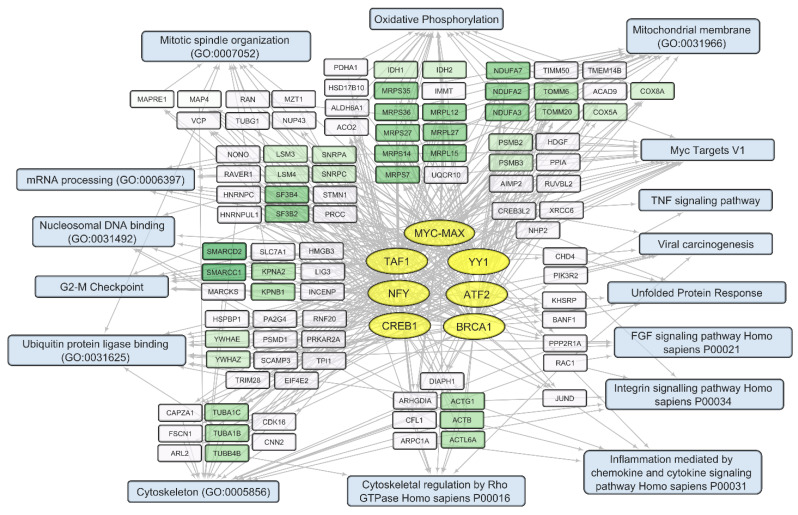
Human transcripts that negatively correlate with the total *VV-GMCSF-Lact* RNA in glioma and NB cells. The scheme illustrates the relationship between the activity of transcription factors of the MYC-MAX family, YY1, ATF2, BRCA1, CREB1, NFY family, and TAF1 (yellow ovals) and transcripts that negatively correlate with the total number of viral RNA reads (white and green rectangles). The associated signaling pathways, biological processes, and other gene annotations are shown in blue rectangles. Groups of genes with similar functions are drawn together using shades of green based on the analysis of gene sets using the Enrichr libraries “ENCODE and ChEA Consensus TFs from ChIP-X”; “MSigDB Hallmark 2020”; “GO Biologic Process 2021”; and “KEGG 2021 Human”.

Notably, some transcription factors (ATF2, CREB1, TAF1, and YY1) can control non-overlapping sets of genes that correlate positively or negatively with the total level of viral RNA (Table S6). This is likely due to the well-known fact that viral transcription factors modulate gene expression by replacing or regulating the activity of host cell transcription machinery [34,59]. From this point of view, host cell transcription factors, which are influenced by the virus and induce both the activation and suppression of transcription in infected cells, can be considered the primary targets of viral transcriptional regulators. The diversity of transcription factors correlating directly or inversely with the level of viral RNA in human cells (Tables S6 and S7) is summarized in Figure 8 and Figure 9, together with the corresponding processes and pathways.

Among human transcripts that correlate positively with viral RNA, histone gene products should be listed separately (Table S7). Histone gene aliases are not uniformly represented in the Enrichr libraries and, therefore, are included in a separate row in Table S7. All the listed histone gene transcripts are present not only among mRNAs that positively correlate with the level of viral RNA (Table S7) but also within the sets of DEGs that are commonly found in infected glioma cells (Table 2). Thus, histone gene transcripts are usually upregulated upon infection of glioma by *VV-GMCSF-Lact* and their relative level can reflect the “depth” of infection corresponding to the amount of viral RNA in cells.

One of the most significant groups of genes that positively correlate with viral RNA is “Herpes simplex virus 1 infection” (Table S7, Figure 8). This group of transcripts indicates the cellular response characteristic of infection with double-stranded DNA viruses, which include members of the Herpes virus family and VACV. Additionally, there are groups of genes encoding proteins that are involved in mRNA processing and regulation of RNA splicing: Cdc2-like protein kinases CLK1, −2, 4; RNA splicing factors SRSF10 and SRSF11; components of the spliceosome CWC22 and CWC25; and pre-mRNA splicing factors RBM5 and RBM39 (Table S7, Figure 8).

Among genes whose expression correlates inversely with the level of viral RNA, it is necessary to single out the groups encoding mitochondrial proteins. These are the components of mitochondrial ribosome proteins (MRPS and MRPL groups of genes) and mitochondrial membrane proteins (Isocitrate Dehydrogenase 2 IDH2; Ubiquinone Oxidoreductase Subunits NDUFA2, −3, −7; Cytochrome C Oxidase Subunit COX5A and COX8A; and others involved in oxidative phosphorylation). Genes encoding components of the cytoskeleton and its regulation are also worth noting. These include tubulins TUBA1C, TUBA1B, and TUBB4B and actins ACTB, ACTG1, and ACL6A. An increase in viral transcripts is accompanied by a downregulation of genes involved in the mitotic spindle organization and the G2/M checkpoint (and others in Table S7, Figure 9), which aligns with the groups described as commonly suppressed genes (Table S5, Figure 6).

Thus, an increase in the level of *VV-GMCSF-Lact* RNA in glioma and NB cells is accompanied by the transcriptional activation of histones, genes involved in post-transcriptional RNA processing, and genes associated with inflammation through chemokine and cytokine signaling pathways, apoptosis, etc. (Table S7 and Figure 8). Host genes whose expression is suppressed with an increase in the level of *VV-GMCSF-Lact* RNA encode mitochondrial proteins, cytoskeletal proteins, and cell cycle regulators at the mitotic spindle organization stage and the G2/M checkpoint (Figure 9, Table S7).

### 3.5. Human Transcripts That Correlate with the VV-GMCSF-Lact Cytotoxic Dose CD_50_ in Glioma and NB Cells

One of the most intriguing questions regarding *VV-GMCSF-Lact* infection is the resistance/susceptibility of human cells to the cytotoxic effects of the virus. Therefore, we analyzed sets of human transcripts whose expression correlates directly or inversely with the cytotoxic dose of *VV-GMCSF-Lact* (CD_50_, Table 1). When the relative level of human mRNAs correlates directly with CD_50_, they could perhaps be considered as transcripts associated with the cell’s resistance to the cytotoxic effect of the virus. Similarly, mRNAs that correlate inversely with CD_50_ might be linked to processes mediating the cell’s sensitivity to the cytotoxic effect of *VV-GMCSF-Lact* (Figure S4).

Transcripts of *VV-GMCSF-Lact*-infected glioma and NB cells that directly correlate with CD_50_ are enriched with mRNAs controlled by transcription factors ATF2, BRCA1, CREB1, ELF1, TAF1, UBTF, and YY1. The sets of transcripts that inversely correlate with CD_50_ are enriched with gene products controlled by the MYC-MAX, E2F, and NFY families of transcription factors, as well as by BRCA1, CREB1, TAF1, and YY1 (Table S8). BRCA1, CREB1, TAF1, and YY1 are the transcription factors that control both sets of genes, i.e., those that either increase or decrease their expression in correlation with the level of the virus RNA (Table S6). Moreover, as in the case of the transcripts correlating with the viral RNA level (Table S6), sets of mRNAs that correlate positively and negatively with CD_50_ are enriched with those controlled by a similar set of transcription factors (BRCA1, CREB1, TAF1, and YY1; Table S8). Considering that during infection, the transcription of human genes is significantly modulated by viral factors [34,59], it can be expected that the gene enrichment analysis shows multidirectional changes in the activity of most affected host cell transcription factors. Importantly, the Enrichr library “ENCODE and ChEA Consensus TFs from ChIP-X” does not include patterns of the genes targeted by viral transcription factors/modulators. Therefore, the action of the viral transcription factors manifests through a combination of the Enrichr library tabulated human TFs.

Downregulated mRNAs common to glioma and NB cells are enriched in transcripts controlled by transcription factors of the E2F and NFY families (Table S4). A decrease in the activity of MYC-MAX and E2F suggests the suppression of human genes with an increasing level of viral RNA (Table S6). The MYC-MAX, E2F, and NFY families of transcription factors are associated with the sensitivity of cells to the cytotoxic effect of the virus (Table S8). Thus, it can be assumed that *VV-GMCSF-Lact* infection proceeds with the suppression of E2F and NFY, while the decrease in MYC-MAX and E2F activity is directly related to the increase in viral transcripts. The decreased activity of all three families, MYC-MAX, E2F, and NFY, is linked to the sensitivity of cells to the cytotoxic effect of the virus (Table S8).

**Figure 10:**
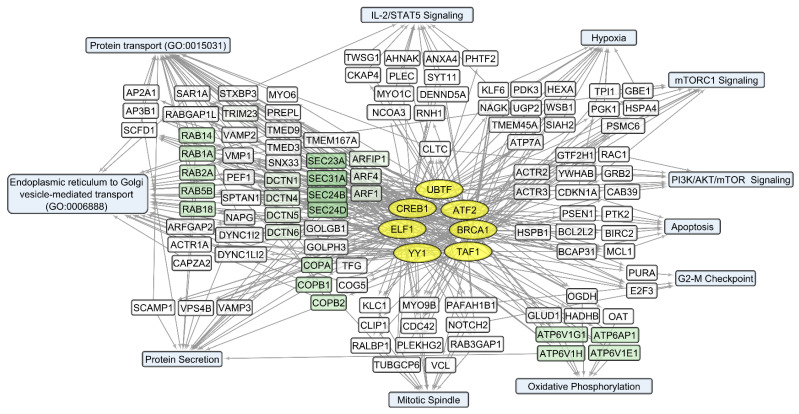
Transcripts that positively correlate with the *VV-GMCSF-Lact* CD_50_. The scheme illustrates the relationship between the activity of transcription factors UBTF, ATF2, BRCA1, TAF1, YY1, ELF1, and CREB1 (yellow ovals) and the corresponding mRNAs whose expression positively correlates with the *VV-GMCSF-Lact* CD_50_ (white and green rectangles). The associated signaling pathways, biological processes, and other gene annotations are shown in blue rectangles. Groups of genes with similar functions are drawn together using shades of green based on the analysis of gene sets using the Enrichr libraries “ENCODE and ChEA Consensus TFs from ChIP-X”; “MSigDB Hallmark 2020”; “GO Biologic Process 2021”; and “KEGG 2021 Human”.

Among biological processes and pathways involving genes whose expression positively correlates with the *VV-GMCSF-Lact* CD_50_ (Table S9), the following are distinguished: a group of small RAB GTPases (RAB1A, RAB2A), regulators of intracellular membrane trafficking; components of the coat protein complex II, which promotes transport vesicle generation from the endoplasmic reticulum (SEC24B, SEC31A); vesicular coat proteins (COPA, COPB1, and COPB2); and dynactins (DCTN1, 4–6) that regulate the transport of vesicles and organelles along microtubules (summarized in Figure 10).

**Figure 11:**
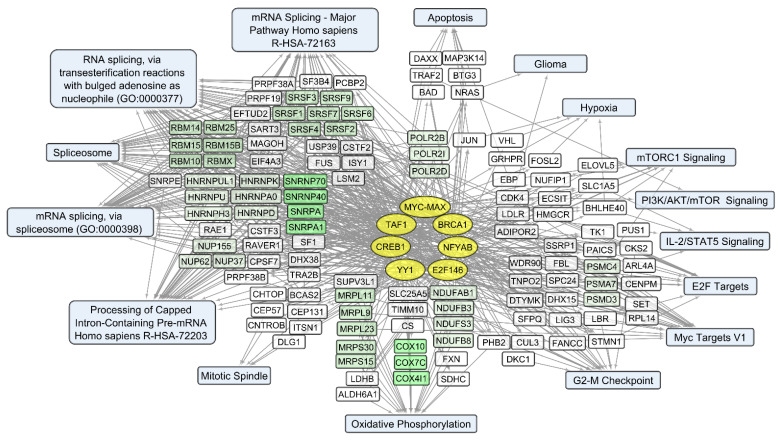
Transcripts that negatively correlate with the *VV-GMCSF-Lact* CD_50_. The scheme illustrates the relationship between the activity of transcription factors of the MYC-MAX family (MYC and MAX), BRCA1, the NFY family (NFYA and NFYB), the E2F family (E2F1, E2F4, and E2F6), YY1, CREB1, and TAF1 (yellow ovals), and the corresponding mRNAs whose expression inversely correlates with the *VV-GMCSF-Lact* CD_50_ (white and green rectangles). The associated signaling pathways, biological processes, and other gene annotations are shown in blue rectangles. Groups of genes with similar functions are drawn together using shades of green based on the analysis of gene sets using the Enrichr libraries “ENCODE and ChEA Consensus TFs from ChIP-X”; “MSigDB Hallmark 2020”; “GO Biologic Process 2021”; and “KEGG 2021 Human”.

Negative correlations with the *VV-GMCSF-Lact* CD_50_ can be observed within several functionally related groups (Table S9, Figure 11). These encode mitochondrial ribosomal proteins (MRPL9, MRPS15); mitochondrial cytochrome c oxidase subunits COX10, COX7C; and mitochondrial respiratory chain complex I—NADH:ubiquinone oxidoreductase subunits (NDUFAB1, NDUFB3). Additionally, there are genes involved in the processing of pol II transcripts: SRSF encoding the processing and regulation of RNA splicing (RNA splicing factors SRSF1, −2, −3); heterogeneous nuclear ribonucleoproteins HNRNPD, HNRNPA0; small nuclear ribonucleoproteins SNRPA, SNRPA1; transcription and splicing regulators RBM10, −14, −15; and others (Table S9, Figure 11).

Taken together, the data on the correlation of human transcript levels with the *VV-GMCSF-Lact* CD_50_ emphasize that the resistance of glioma and NB cells to viral infection is largely associated with the activity of genes encoding components of endoplasmic reticulum, intracellular transport, and secretion. The susceptibility of cells to viral cytotoxicity is associated with the activity of human genes encoding mitochondrial proteins and factors of nuclear RNA processing and splicing.

### 3.6. Viral Transcripts That Correlate with the Cytotoxic Dose of the Virus in Glioma and NB Cells Infected with VV-GMCSF-Lact

The search for mRNAs expressed in correlation with the cytotoxic dose applies to both human and viral genome transcripts. Therefore, we analyzed the correlation between the viral CD_50_ values and the levels of viral transcripts in *VV-GMCSF-Lact*-infected glioma and NB cells. No viral mRNAs correlate positively with CD_50_ (Pearson R > 0.5). This suggests that none of the *VV-GMCSF-Lact* transcripts are associated with processes providing cellular resistance to the cytotoxic effects of the virus.

**Figure 12:**
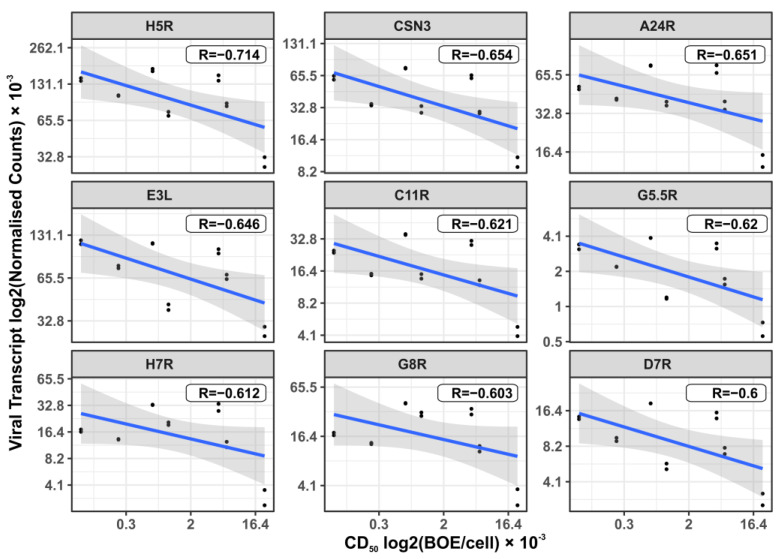
Dot plots representing negative correlations of *VV-GMCSF-Lact* transcripts with the virus cytotoxic dose (CD_50_) in glioma and NB cells 24 h after infection. R—Pearson’s correlation coefficient. Viral gene’s box plots sorted from upper left to lower right by increasing R. The plots are sorted from top left to bottom right based on the increasing Pearson correlation coefficient R, ranging from the highest inverse correlation to the lowest.

Among the *VV-GMCSF-Lact* transcripts that inversely correlate with CD_50_ and are thus associated with its cytotoxicity, the following stand out: H5R (late transcription factor), A24R (VLTF-4 DNA-dependent RNA polymerase subunit rpo132), and E3L (double-strand RNA-binding protein). H5R, A24R, and E3L are also detected as commonly expressed transcripts (Table 3). Importantly, the level of viral CSN3 RNA negatively correlates with the *VV-GMCSF-Lact* CD_50_ in glioma and NB cells, ranking second (R = −0.654) after the viral H5R transcript R = −0.714, Figure 12).

Thus, the cytotoxicity of *VV-GMCSF-Lact* against glioma and NB cells results from both VACV genome-encoded factors and modifications to the viral genome by the human CSN3 gene fragment encoding the proapoptotic peptide lactaptin.

## 4. Discussion

Oncolytic virotherapy is a rapidly evolving approach that aims to selectively kill cancer cells while leaving normal, non-cancerous cells viable. Vaccinia virus (VACV) is one of the most explored platforms for creating oncolytic treatments for various malignant neoplasms [60]. Vaccinia is a dsDNA virus of the *Poxviridae* family. Its DNA (~195 kb) encodes approximately 250 genes [34] and, together with proteins and lipids, forms a ~360 nm × 270 nm × 250 nm virion [44]. The virus enters cells via either micropinocytosis or direct fusion with the plasma membrane [61]. The infection is accompanied by a global change in the molecular and genetic landscape of the cell. It can lead to either the suppression of the virus or the death of the infected cell. A previous study using deep RNA sequencing on VACV-infected cervical cancer Hela cells identified upregulated host cell RNAs linked to the NF-κB cascade, apoptosis, signal transduction, and ligand-mediated signaling, likely representing the response to the virus invasion [62].

Recently, we developed a recombinant VACV named *VV-GMCSF-Lact* as a carrier for creating drugs for the treatment of malignant tumors, including glioblastoma. To construct *VV-GMCSF-Lact* from the original L-IVP strain, the viral thymidine kinase (tk) gene was replaced with the human GM-CSF, and the viral growth factor (vgf) gene was replaced with a fragment of the human CSN3 gene encoding the proapoptotic peptide lactaptin [11]. Here, we applied NGS to analyze the effect of *VV-GMCSF-Lact* infection on the transcriptome of human glioma and normal brain cells in culture (Tables S1 and S2). In terms of the sample tree and PCA, *VV-GMCSF-Lact* infection induced significant transcript changes in all analyzed cell cultures (Figure 3). The number of differentially expressed human genes correlated with the relative amount of viral transcripts (Figure S2). Thus, the data allowed us to describe trends in viral infection, covering both general and cell culture-specific patterns. To overview the effect of *VV-GMCSF-Lact* on cells, we analyzed sets of transcripts that are typically up/downregulated in infected cells, exhibit positive or negative correlations with the viral RNA level in cells, or are associated with the virus cytotoxic dose (CD_50_).

Among human transcripts that are commonly upregulated following *VV-GMCSF-Lact* infection, special attention should be given to mRNA of histone genes. Transcripts representative of all major histone families (H1/H5, H2A, H2B, H3, and H4) are either generally activated upon infection or correlate positively with the total viral RNA levels in glioma and NB cells (Figure 4, Table 2 and Table S5). This aligns with data from the literature, as activation of histone gene expression is also observed upon infection of *Macaca mulatta* kidney epithelial cells with another member of the *Poxviridae* family, the *Monkeypox virus* [63,64].

It is known that double-stranded VACV DNA is replicated in cytoplasmic structures [65], and cellular proteins such as histones are not involved in VACV genome organization [34]. If present in viral factories, histones are not directly involved in the life cycle of the virus. Therefore, we should not assume that histones interact with viral DNA, even though the transport/exchange of proteins between the cytoplasmic viral factories and the nucleus has been shown for viral proteins [46,66]. However, it can be hypothesized that the activation of histone genes induced by *VV-GMCSF-Lact* reflects the modulation of nuclear processes, including nucleosome organization, mitotic spindle organization, and the G2/M checkpoint in particular (Tables S5 and S7, Figure 4). The upregulation of histone genes may result from the viral factors affecting nuclear DNA compaction and, in general, from the suppression of the cell cycle of an infected cell. This is indirectly confirmed by data indicating the involvement of the VACV K7 protein in histone methylation [67] and the C6 protein in proteasomal degradation of histone deacetylase [68].

Transcripts commonly downregulated during *VV-GMCSF-Lact* infection and showing negative correlation with total viral RNA in glioma and NB cells are enriched in mRNA of genes that regulate processes like the mitotic spindle organization, mitotic sister chromatid segregation, the G2/M checkpoint, and other cell cycle-related functions. The activity of genes controlling vital processes such as cytoskeletal function, mitochondrial translation, and oxidative phosphorylation is also suppressed upon the infection (Tables S5 and S7, Figure 6 and Figure 9). Thus, *VV-GMCSF-Lact* infection significantly modulates nuclear processes, redirecting them to the inhibition of cellular DNA replication, cell cycle arrest, and the suppression of vital cytoplasmic and mitochondrial pathways. Moreover, genes of the antiviral inflammatory response are activated in infected glioma and NB cells. These include groups of transcripts that mediate a defense response to the virus: interferon-gamma response, NF-kappa B signaling pathway, inflammation mediated by chemokine and cytokine signaling pathways, and others (Figure 5, Table S5). This is partly consistent with previous studies of the transcriptome of VACV-infected HeLa cells [62]. Overall, our results and data from the literature point to the competition between virus-induced suppression of cell viability and the host antiviral response to infection.

To understand what processes are responsible for the cellular resistance or vulnerability to the cytotoxic effect of *VV-GMCSF-Lact*, we analyzed the correlation between the levels of individual cellular RNAs of glioma and NB cells with the CD_50_ cytotoxic dose of the virus. Transcript groups that show positive correlations with CD_50_ and that are thus are associated with cellular resistance to viral cytotoxicity encode gene products involved in protein transport, protein secretion, and the endoplasmic reticulum to Golgi vesicle-mediated transport (Table S9, Figure 11).

It is known that the life cycle of VACV begins in the plasma membrane. The replication of the vaccinia virus occurs in the cytoplasmic crescent-shaped vesicle-like structures, resembling mini-nuclei, which originate from the smooth endoplasmic reticulum (“viral factories” [69]). Both viral and cellular proteins move between viral factories and the host cytoplasm [65]. The synthesis and assembly of VACV particles relies on the host’s translational apparatus and protein transport occurring between the endoplasmic reticulum and the Golgi apparatus (reviewed in [59]). Our data suggest that glioma and NB cells are less vulnerable to the cytotoxic effect of *VV-GMCSF-Lact* due to increased expression of the genes of the RAB, DCTN, SEC, and COPA families, which encode the components of Golgi vesicles, protein transport, and secretion (Figure 10, Table S9).

Important gene clusters, whose expression correlates negatively with the *VV-GMCSF-Lact* CD_50_, encode proteins from the SR, RBM, SNRP, HNRNP, and NUP families, which participate in the maturation of pol II nuclear transcripts and mRNA splicing (Table S9, Figure 11). The VACV genes lack introns, and their transcription is not thought to require the nuclear apparatus for mRNA splicing [70]. The decapping of transcripts from intron-containing genes helps the virus to deplete host transcripts and remodel the infected cell transcriptome [71]. Huang et al. showed that in VACV-infected human cells, SR proteins are hypophosphorylated and functionally inactivated as splicing repressors or splicing enhancers [72]. These data indicate that VACV infection represses post-transcriptional processing and modulates the nuclear-cytoplasmic transport of pol II transcripts in the host cell. Considering our data on the negative correlation of human cell transcripts with the *VV-GMCSF-Lact* CD_50_ (Table S9, Figure 11), it can be proposed that an upregulation of genes that control mRNA splicing and processing of capped intron-containing pre-mRNAs represents one of the main factors of cell sensitivity to the cytotoxic effect of the virus.

Genes showing a positive or negative correlation with CD_50_ may be of interest for developing targeted drugs aimed at enhancing the effectiveness of virotherapy (Tables S8 and S9, Figure 10 and Figure 11). For example, by knocking down genes whose expression correlates positively with CD_50_, one can expect an increase in the sensitivity of tumor cells to the virus. A similar effect could be promoted by stimulating the expression of genes that inversely correlate with CD_50_.

Among the viral transcripts that are commonly expressed in *VV-GMCSF-Lact*-infected glioma and NB cells, there is E3L, encoding a dsRNA-binding protein involved in the inhibition of innate immune responses; E9L, the catalytic subunit of viral DNA polymerase; I3L, an ssDNA-binding protein; I4L, the ribonucleotide reductase large subunit; and CSN3, or lactaptin, the fragment of human kappa-casein gene inserted in the *VV-GMCSF-Lact* genome (Table 3). This suggests that our *VV-GMCSF-Lact* viral construct is a powerful vector for the expression of genes in human cells. It is noteworthy that viral CSN3 mRNA is the second most important transcript (after VACV H5R) showing a negative correlation with CD_50_, as it is directly related to the cytotoxic effect of the virus on glioma cells (Figure 12). These data indicate that the cytotoxicity of the *VV-GMCSF-Lact* construct against glioma cells is mediated by both vaccinia virus genes and the recombinant gene encoding the human proapoptotic peptide lactaptin.

Overall, our study links the activity of transcription factors and their target genes to the cellular processes and signaling pathways involved in *VV-GMCSF-Lact* infection of glioma and NB cells. The list of transcription factors modulated by the infection includes the IRF, MYC-MAX, NFY, and E2F families; RELA; YY1; CREB1; and others (Tables S4, S6, and S8; Figure 5, Figure 6, Figure 8, Figure 9, Figure 10 and Figure 11). To describe these transcription factors, we compiled a brief review of data from the literature on their known properties, targets, and interactions (Table S10).

Taken together, our data, highlighting transcription factors, molecular markers, biological pathways, and networks involved in glioma cell sensitivity to *VV-GMCSF-Lact*, can be used to improve the effectiveness of cancer virotherapy.

## 5. Conclusions

As one of the most extensively studied viral construct platforms, VACV has been explored for developing oncolytic therapy against various malignant neoplasms, including glioblastoma. Here, we describe the impact of a VACV-based viral construct, *VV-GMCSF-Lact*, on the transcriptomes of patient-derived glioma/glioblastoma cell cultures, immortalized human glioblastoma cells, and a culture of normal, non-malignant brain cells. For the first time, we conducted a thorough correlation analysis of the human transcriptomic data with viral RNA and the cytotoxic dose (CD_50_).

Both common and specific transcriptome changes indicate that mRNAs representing all major histone families are generally upregulated during infection with *VV-GMCSF-Lact*. They show positive correlations with viral RNA levels in glioma and NB cells. Genes of the antiviral inflammatory response are also activated in glioma and NB cells.

Conversely, genes involved in mitosis, the G2/M checkpoint, and other cell cycle-related functions are downregulated upon infection, and their transcripts correlate negatively with total viral RNA. The activity of genes controlling vital cellular processes, such as cytoskeletal function, mitochondrial translation, and oxidative phosphorylation, is also downregulated during the *VV-GMCSF-Lact* infection.

*VV-GMCSF-Lact* expresses the human CSN3 mRNA fragment encoding the proapoptotic peptide lactaptin. It is directly associated with the cytotoxic effect of the virus on glioma cells, once again affirming the rationale behind selecting this specific gene for insertion into the vector.

To identify RNA groups that are associated with cellular resistance or susceptibility to the cytotoxic effects of *VV-GMCSF-Lact*, we analyzed a correlation between individual transcripts in infected cells and the cytotoxic dose of the virus. Greater resistance to the cytotoxic effect of *VV-GMCSF-Lact* is observed in cells with the increased expression of genes encoding components of Golgi vesicles, protein transport, and secretion. In contrast, cells that are more sensitive to virus cytotoxicity demonstrate the upregulation of genes encoding proteins involved in the maturation of pol II nuclear transcripts and mRNA splicing. Notably, genes whose expression correlates positively or negatively with CD_50_ may be of interest for developing targeted drugs that increase the effectiveness of virotherapy. In conclusion, our data generalize the responses of glioma cells to the oncolytic virus infection and provide insight into the associated molecular pathways. These findings can be used to improve the effectiveness of virotherapy against malignant neoplasms.

## Figures and Tables

**Table 1:** Immortalized human brain cell lines, patient-derived cell cultures, and their response to *VV-GMCSF-Lact* infection.

Cell Culture	Diagnosis ^(1)^	Tumor Grade ^(1)^	Cell Markers (%)					Cell Index ^(2)^	CD_50_^(3)^ (PFU/Cell)	Apoptotic Markers ^(4)^ (%)	
			CD133+	CD44+	CD133+/CD44+	CD171+	CD15+			AnnV+/PI-	AnnV+/PI+
BR1	GBM	IV	15.1	67.7	14.2	24.03	0	11.12	6.3 × 10^−3^	31.3	57.7
BR3	GBM	IV	3.83	90.9	3.69	0	0	3.43	2.4 × 10^−2^	23.6	6.92
BR4	GBM	IV	9.92	78.9	9.56	20.4	1.95	0.94	6.5 × 10^−1^	12.8	5.42
BR5	Anaplastic astrocytoma	III	0.57	34.7	0.37	0	0	3.45	5.2 × 10^−1^	13.1	17.7
NB	Normal brain	--	0	99.7	0	44.1	0	1.07	5.8 × 10^0^	5.7	0.74
U87	GBM	IV	0	7.47	0	0	50.1	1.39	1.2 × 10^−1^	18.2	8.31
U343	GBM	IV	2.48	15.9	0.59	0	6.04	2.59	6.5 × 10^−2^	35.9	5.63

^(1)^—Brain tumor diagnosis and grade according to the WHO Classification of Tumors of the Central Nervous System 2016. ^(2)^—The cell index represents the measure of cellular adhesion and proliferation across each individual well. ^(3)^—CD_50_ is the dose of *VV-GMCSF-Lact* causing 50% cell death. ^(4)^—The percentage of AnnV+/PI- or AnnV+/PI+ cells in cultures infected with *VV-GMCSF-Lact*, 1 PFU/cell within 24 h.

**Table 2:** Transcripts upregulated or downregulated in all glioma and NB cell cultures upon infection with *VV-GMCSF-Lact*.

Infection Time, Regulation		Number of DEGs	DEGs *
**12 h**	Up	5	**H2BC5; H4C5; H2BC8; H4C8; H4C2**
	Down	1	MARCKS
**24 h**	Up	85	AHSA2P; AOC2; ARID4B; AVIL; AXIN2; B3GNT2; BAZ2A; BRD1; CCDC17; CCNL1; CDKN2AIP; CLCN6; CLDN15; CLK4; CMYA5; CWC22; CWC25; DNAJA1; DNHD1; FGFR1OP2; FLCN; FNBP4; **H1-2; H1-3; H1-4; H2AC17; H2AC4; H2AC6; H2AC8; H2BC11; H2BC15; H2BC18; H2BC21; H2BC4; H2BC5; H2BC8; H2BC9; H3C3; H3C4; H4C2; H4C3; H4C5; H4C8**; HSPA1B; HSPA6; JMJD1C; KDM6A; KHDC4; LIME1; LOC102724814; LOC284454; LOC729603; LSMEM1; LTB4R; MAPK8IP3; MBIP; MORC3; NFKBIZ; NXF1; PAXBP1; PPTC7; PTGS2; PUM2; QRICH2; RBBP6; RBM33; RBM5; RNU4-2; RPPH1; RSRC2; SCARNA2; SERTAD1; SLC25A25; SLC8A2; SMPD4BP; SYNGAP1; TENT4B; TMEM259; UBE2B; WAC; YOD1; ZFYVE27; ZNF160; ZNF211; ZNF451
	Down	54	ACTB; ACTR1A; AP2B1; ARF3; ARPC4; ATP5MC2; C11orf68; CALD1; CAPNS1; CAPRIN1; CAPZB; CAVIN1; CDC42; CDK4; CFL2; CLTA; CNN2; COPZ1; CSRP1; DAZAP2; EHD2; EI24; EPN1; FAM98A; FSCN1; IMMT; LASP1; LOXL1; LRRC59; MAP1A; MAP4; MARCKS; NONO; PABPC4; PARVA; PLIN3; RHOA; RNF20; RSU1; RTL8C; S100A11; SH3BGRL3; SKI; SMARCC1; SNX12; STMP1; TLN1; TRAPPC1; TXN2; UQCR10; UROS; USP22; VIM; WFS1

*—Differentially expressed histone genes are highlighted in **bold**.

**Table 3:** Top 25 *VV-GMCSF-Lact* transcripts commonly expressed in glioma and NB cells. Viral transcripts were ranked in descending order based on DESeq2 log2FoldChange, combining data from both the 12 h and 24 h time points for each cell line. The top 25 genes with the highest expression are listed after averaging the ranks.

Viral Gene	Average Rank ^(1)^	Temporal Expression ^(2)^	Preferred Name ^(2)^ Annotation	Ref
E3L	30.1	Early	Double-strand RNA-binding protein. Host range function and inhibition of innate immune responses.	[36]
E9L	33.3	Early	DNA polymerase. The catalytic subunit, a family B DNA polymerase.	[37]
I3L	34.1	Early Intermediate Late	Single-stranded DNA-binding protein.	[38]
I4L	34.8	Early	Ribonucleotide reductase large subunit.	[39]
B8R	35.1	Early	Soluble interferon-gamma receptor-like protein. Host defense modulator.	[40]
H5R	35.9	Early Late	Late transcription factor VLTF-4. DNA synthesis, postreplicative gene transcription, and virion morphogenesis.	[41]
O1L	37.5	--	Hypothetical protein. Positive regulator of the ERK1/2 pathway downstream of the EGFR.	[42]
D1R	38.6	Early	Large subunit of mRNA capping enzyme. Transcription termination factor.	[43]
F4L	39.6	Early	Ribonucleotide reductase small subunit.	[39]
J6R	39.7	Early Late	DNA-dependent RNA polymerase subunit rpo147.	[44]
A12L	40.2	Late	Core protein.	[45]
C4L	41.2	--	Inhibits NF-kB activation and promotes virus virulence	[46]
B2R	42.9	--	Hypothetical protein. Forms part of the envelope of the extracellular virus.	[47]
CSN3	43.4	--	**Lactaptin. Proapoptotic fragment of the human kappa-casein gene, inserted in the VACV genome ^(3)^**	[10]
F1L	44.2	Early	Hypothetical protein. Suppressor of NLR family proteins involved in IL-1β activation.	[48]
D13L	44.4	Late	Rifampicin target. Rifampicin resistance.	[49]
A3L	44.6	Late	P4b precursor of core protein 4b. Critical for proper formation of the core wall and nucleocapsid.	[50]
D5R	44.6	Early Late	NTPase. DNA primase.	[51]
A10L	46.0	Late	Precursor p4a of core protein 4a.	[52]
A37R	46.5	--	Hypothetical protein. Regulate the release of cell-associated virions.	[53]
A24R	47.9	Early Late	DNA-dependent RNA polymerase subunit rpo132.	[54]
F12L	48.3	Early	EEV maturation protein. Facilitates the transport of intracellular viral particles to the cell membrane.	[55]
A46R	48.4	--	Toll/IL1-receptor. Antagonists of the activation of the proinflammatory transcription factor NF-κB.	[56]
A35R	49.4	--	Hypothetical protein. Inhibitor of MHC class II-restricted antigen presentation.	[57]
M1L	50.2	Early	Ankyrin-like protein. Apoptosis inhibitor	[58]

^(1)^—Average rank of a viral transcript in glioma and NB cells (both 12 h and 24 h of infection) sorted in descending order. ^(2)^—Temporal expression and preferred names from the NCBI Gene database (https://www.ncbi.nlm.nih.gov/gene/). ^(3)^—Transcript of the human CSN3 gene fragment inserted into the genome of VACV is highlighted in bold.

## Data Availability

RNA-seq data from seven glioma cell cultures infected with VV-GMCSF-Lact have been deposited in the SRA database—accession code PRJNA1031253, with the exception of data from control cultures BR1, U343 and U87 not infected with the virus, which were previously deposited under accession code PRJNA869596 (series BR1a, U343a and U87a, respectively). All other data supporting the findings of this study are available in the article and supplementary files.

## Supplementary Materials

The following supporting information can be downloaded at: https://www.mdpi.com/article/10.3390/cells12222616/s1, Table S1: Characteristics of high-throughput RNA sequencing data arrays of immortalized and patient-derived human brain cell cultures at different time points during infection with *VV-GMCSF-Lact*; Table S2: Number of human RefSeq annotated transcripts (differentially expressed genes, DEGs) altered by *VV-GMCSF-Lact* infection of glioma and NB cell cultures (1 PFU per cell); Figure S1: Selected differentially expressed genes (DEGs) of human glioma and NB cells infected with *VV-GMCSF-Lact* (1 PFU per cell). Representative box plots show changes in the relative amounts of selected upregulated or downregulated mRNAs common to the analyzed cell cultures upon infection with *VV-GMCSF-Lact*. All shown genes meet the condition of DESeq2 comparisons: padj < 0.01 for 0 h vs. 24 h; Figure S2: Correlation between the relative contribution of viral transcripts and the number of differentially expressed genes (DEGs) in glioma cells and NB cells infected with *VV-GMCSF-Lact* (1 PFU per cell). The proportion of viral transcripts was determined as the ratio of viral DNA-mapped RNA-Seq reads to human genome-mapped reads. The dotted line represents a linear approximation of the dependence between the number of DEGs and the relative proportion of viral transcripts. R2 is the squared Pearson’s coefficient of the linear correlation; Table S3. The proportions of common and unique human cell transcripts that are upregulated or downregulated upon *VV-GMCSF-Lact* infection (1 PFU per cell); Table S4: Common transcription factors involved in the response of glioma and NB cells to *VV-GMCSF-Lact* infection. The table includes selected results of the Enrichr analysis of the top 300 transcripts that are upregulated (Up) or downregulated (Down) during infection with 1 PFU of the virus per cell (Enrichr terms of “ENCODE and ChEA Consensus TFs from ChIP-X” library); Table S5: Common biological processes and pathways affected upon infection of glioma and NB cells with *VV-GMCSF-Lact*. Selected Enrichr annotations are presented, common for the top 300 upregulated or downregulated differentially expressed genes identified in more than five out of seven cell cultures at 12 h and 24 h post-infection; Figure S3: Selected human transcripts that correlate directly or inversely with the total viral RNA in glioma and NB cells infected with *VV-GMCSF-Lact*. Dot plots show correlations between the expression of human transcripts and the normalized count of *VV-GMCSF-Lact* transcripts. R – Pearson’s correlation coefficient; Table S6: Transcription factors involved in regulating genes whose expression correlates with the total number of viral transcripts in glioma and NB cells infected with *VV-GMCSF-Lact*. The table presents the results of Enrichr analysis of the top 300 transcripts showing direct or inverse correlation with the total *VV-GMCSF-Lact* mRNA (Pearson coefficient R > 0.5 for direct, and R < – 0.5 for inverse correlation). Selected Enrichr terms of the “ENCODE and ChEA Consensus TFs from ChIP-X” library are listed; Figure S4: Dot plots of the correlations between the expression of human transcripts and the *VV-GMCSF-Lact* cytotoxicity index (CD_50_) 0, 12, or 24 h post-infection. Selected human transcripts, which correlate directly or inversely with the cytotoxicity index (CD_50_) of *VV-GMCSF-Lact* in glioma and NB cells, are shown. R – Pearson’s correlation coefficient; Table S7: Biological processes and pathways that are associated with gene sets correlating with the total number of viral transcripts in *VV-GMCSF-Lact*-infected human glioma and NB cells. Based on the Enrichr analysis of the top 300 transcripts that correlate directly or inversely with *VV-GMCSF-Lact* mRNA (Pearson coefficient R > 0.5 for direct, and R < – 0.5 for inverse correlation). Selected Enrichr terms from the GO, KEGG, MSigDB Hallmark, and Panther libraries are used; Table S8: Transcription factors regulating genes whose expression directly or inversely correlates with the *VV-GMCSF-Lact* cytotoxic dose (CD_50_). Based on the Enrichr analysis of transcripts sets (top 300 according to Pearson R) that directly or inversely correlate with CD_50_ 0, 12, or 24 h post-infection (Pearson coefficient R > 0.5 for direct, and R < – 0.5 for inverse correlation). Enrichr terms of the “ENCODE and ChEA Consensus TFs from ChIP-X” library are used; Table S9: Selected biological processes and pathways associated with sets of human genes whose expression correlates with the cytotoxic dose of *VV-GMCSF-Lact* (CD_50_). Based on the Enrichr analysis of transcripts sets (top 300 according to Pearson R) that directly or inversely correlate with CD_50_ 0, 12, or 24 h of infection (Pearson coefficient R > 0.5 for direct, and R < – 0.5 for inverse correlation). Enrichr terms of the GO, KEGG, MSigDB Hallmark, and Reactome libraries are used; Table S10: A brief review of literature data on properties, targets, and interactions of human transcription factors modulated by *VV-GMCSF-Lact*.

## Abbreviations

CD_50_	cytotoxic dose
CI	cell index
CSN2	kappa-casein gene
DEG	differentially expressed gene
GBM	glioblastoma, previously referred to as “glioblastoma multiforme”
GM-CSF	granulocyte-macrophage colony-stimulating factor (product of CSF2 gene)
HC	hierarchical clustering
MOI	the multiplicity of infection
NB	normal brain tissue cell culture
PCA	principal component analysis
PFU	plaque-forming unit
tk	thymidine kinase
U343	U343 MG cell culture
U87	U87 MG cell culture
VACV	vaccinia virus
vgf	virus growth factor
VST	variance stabilizing transformation
