# Supplementary material for: Transcriptome Changes in Glioma Cells upon Infection with the Oncolytic Virus VV-GMCSF-Lact
Source: Cells. 2023 Nov 12;12(22):2616. doi: 10.3390/cells12222616 (PMC10670333; doi:10.3390/cells12222616)
Supplement: Supplementary file 1 [file cells-12-02616-s001.zip › cells-2656698-supplementary.pdf]

Table S1. Characteristics of high-throughput RNA sequencing data arrays of immortalized and patient-derived human brain cell cultures at different time points during infection with *VV-GMCSF-Lact*.

Cell culture	Histological type	Time of infection (h)	NGS library	Number of replicates	Sequencing depth (mln reads)
**BR1**	GBM*	0	BR1_K	2	22.94
		12	BR1_12H	2	35.20
		24	BR1_24H	2	37.49
**BR3**	GBM	0	BR3_K	2	19.55
		12	BR3_12H	2	36.89
		24	BR3_24H	2	37.95
**BR4**	GBM	0	BR4_K	2	24.21
		12	BR4_12H	2	34.11
		24	BR4_24H	2	34.04
**BR5**	Anaplastic astrocytoma	0	BR5_K	2	41.93
		12	BR5_12H	2	38.62
		24	BR5_24H	2	38.31
**NB**	Normal brain	0	NB_K	2	39.34
		12	NB_12H	2	38.20
		24	NB_24H	2	39.28
**U343**	GBM	0	U343_K	4	46.60
		12	U343_12H	2	23.97
		24	U343_24H	2	24.80
**U87**	GBM	0	U87_K	4	46.35
		12	U87_12H	2	21.79
		24	U87_24H	2	23.45

*– GBM – glioblastoma

Table S2. Number of human RefSeq annotated transcripts (differentially expressed genes, DEGs) altered by *VV-GMCSF-Lact* infection of glioma and NB cell cultures (1 PFU per cell).

Cell Culture	Infection Time (h)	Contribution of Viral Reads (%)	Number of human DEGs*		
			Total	Upregulated	Downregulated
BR1	12	22.12	5262	2735	2527
	24	35.59	6587	3465	3122
BR3	12	24.47	3043	1483	1560
	24	30.44	2814	1365	1449
BR4	12	14.91	2691	1240	1451
	24	26.50	3854	1783	2071
BR5	12	16.35	856	468	388
	24	60.18	6209	3238	2971
NB	12	5.290	904	484	420
	24	7.850	1563	977	586
U343	12	11.16	258	157	101
	24	73.46	6158	3185	2973
U87	12	3.040	1030	510	520
	24	38.21	1384	697	687

* – The transcripts are considered DEGs if their normalized level meets the conditions DESeq2: 0 h vs 12 or 0h vs 24 h after *VV-GMCSF-Lact* infection, with padj < 1.0E-3 and log2FoldChange < 0 for downregulated, or padj < 1.0E-3 and log2FoldChange > 0 for upregulated human genes.

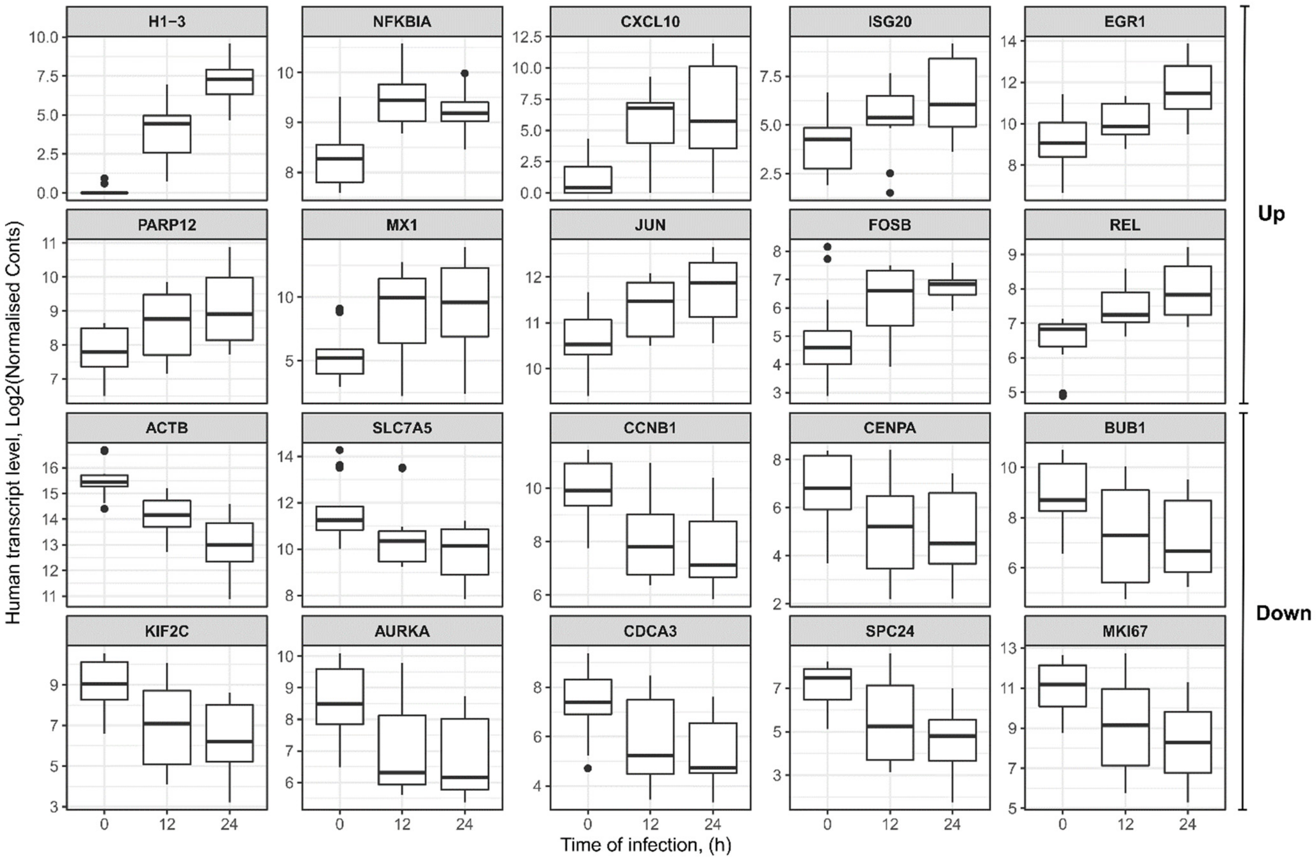

Figure S1. Selected differentially expressed genes (DEGs) of human glioma and NB cells infected with *VV-GMCSF-Lact* (1 PFU per cell). Representative box plots show changes in the relative amounts of selected upregulated or downregulated mRNAs common to the analyzed cell cultures upon infection with *VV-GMCSF-Lact*. All shown genes meet the condition of DESeq2 comparisons: padj < 0.01 for 0 h vs. 24 h.

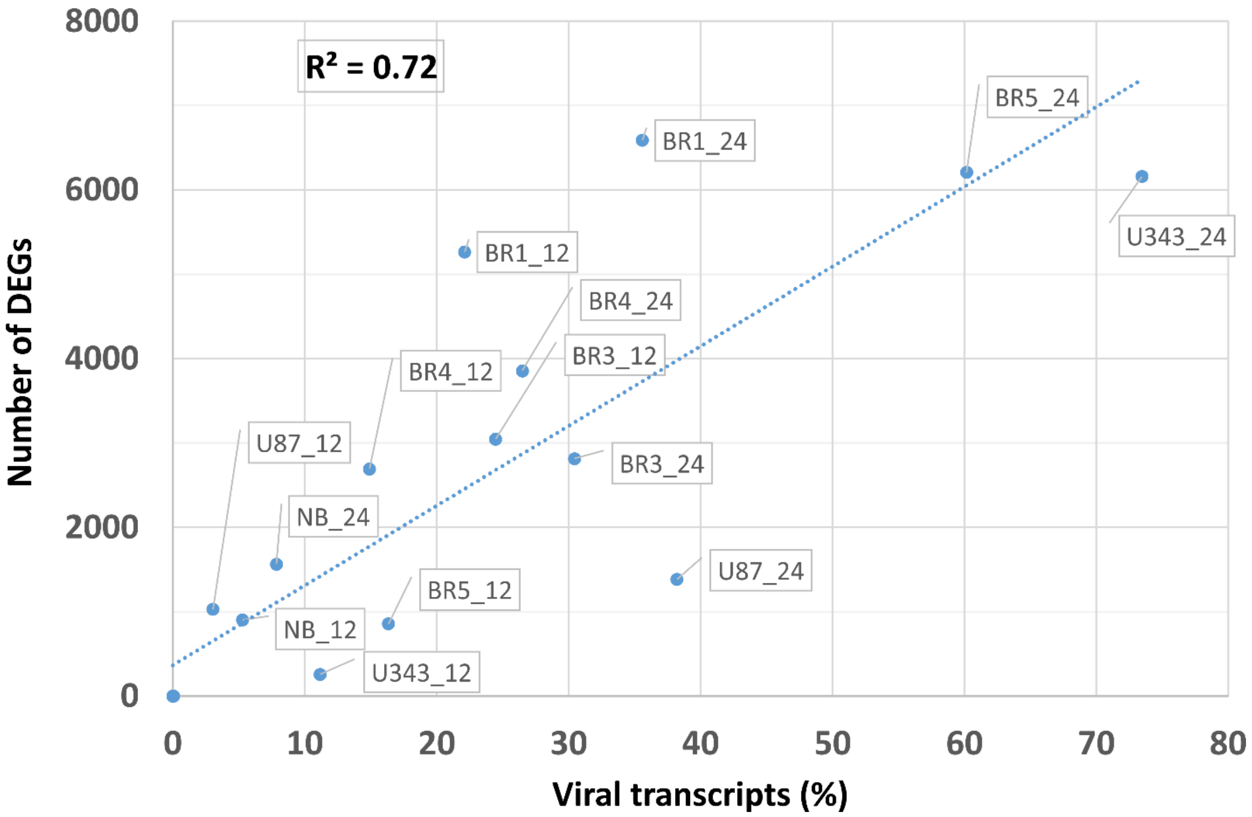

Figure S2. Correlation between the relative contribution of viral transcripts and the number of differentially expressed genes (DEGs) in glioma cells and NB cells infected with *VV-GMCSF-Lact* (1 PFU per cell). The proportion of viral transcripts was determined as the ratio of viral DNA-mapped RNA-Seq reads to human genome-mapped reads. The dotted line represents a linear approximation of the dependence between the number of DEGs and the relative proportion of viral transcripts. R^2 is the squared Pearson's coefficient of the linear correlation.

Table S3. The proportions of common and unique human cell transcripts that are upregulated or downregulated upon *VV-GMCSF-Lact* infection (1 PFU per cell).

**Condition**		**Unique (%)***	**Common (%)****					
			**2**	**3**	**4**	**5**	**6**	**7**
**12 h**	**Up**	65.89	20.70	8.37	3.50	0.98	0.44	0.11
	**Down**	62.92	22.52	9.87	3.49	1.04	0.14	0.02
**24 h**	**Up**	39.99	23.19	17.31	9.67	5.41	3.09	1.34
	**Down**	37.56	24.51	17.93	10.53	5.95	2.60	0.91

* Proportion of transcripts that are found to change their expression in only one specific cell line.
** Proportion of transcripts that are found to change their expression in two or more cell lines.

Table S4. Common transcription factors involved in the response of glioma and NB cells to VV-GMCSF-Lact infection. The table includes selected results of the Enrichr analysis of the top 300 transcripts that are upregulated (Up) or downregulated (Down) during infection with 1 PFU of the virus per cell (Enrichr terms of "ENCODE and ChEA Consensus TFs from ChIP-X" library).

Regulation	Term	TF Group / Family	Representative Genes	Number of genes
Up	IRF8 CHEA	**IRF**	MX2;IFIH1;TNFSF13B;DDX58;ISG20CD274;RTP4;USP18;CTSS;IFI6;ISG15;PARP14	89
	IRF1 ENCODE			
	FOSL2 ENCODE	**FOS**	ISG20;NFATC2;ITGA2;EFNA1;MYH16;NR1D1;TNFAIP3;IGFBP6;ANKRD1	43
	REST ENCODE	**REST**	SLC8A2;GRM2;GOLGA7B;CELF6;CELSR3;COL5A3;ARC;EGR4;GRIP2;EEF1A2	225
	REST CHEA			
	SRF ENCODE	**SRF**	EGR4;EGR3;FOSB;BATF2;MAP3K14;EGR1;TERC;NR4A3;DUSP5;FOS	30
	RELA ENCODE	**NFkB**	IFIH1;BST2;RND1;TIPARP-AS1;RMRP;BIRC3;NFKBIA;NOD2;TNFAIP3;ICAM1	85
Down	NFYA ENCODE	**NFY**	ACTB;AURKB;TK1;KIF20A;RRM2;CDC20;NEK2;SPC24;UCP2;CENPF;MKI67;RRM2	698
	NFYB ENCODE			
	E2F1 CHEA	**E2F**	LMNB1;AURKB;TK1;RRM2;UBE2C;SPC24;BUB1B;CCNB2;G6PD;BIRC5;TK1;KIF20A;IDH2	337
	E2F4 ENCODE			
	E2F6 ENCODE			

Table S5. Common biological processes and pathways affected upon infection of glioma and NB cells with *VV-GMCSF-Lact*. Selected Enrichr annotations are presented, common for the top 300 upregulated or downregulated differentially expressed genes identified in more than five out of seven cell cultures at 12 h and 24 h post-infection.

Enrichr Library	Regulation	Term	Representative Genes	Number of genes
GO Biological Process 2021	Up	Cytokine-mediated signaling pathway (GO:0019221)	H3C1;CXCL10;CXCL11;OASL;RSAD2;H3C13;CCL20;MX1;CXCL3;OAS1	148
		Nucleosome organization (GO:0034728)	H1-3;H1-4;H4-16;H2BC5;H3C1;H1-5;NAP1L2;SART3;ATRX;SOX9	26
		Defense response to virus (GO:0051607)	CXCL10;OASL;RSAD2;MX1;OAS1;IFI44L;IFI6;IFIT2;IFIT1;ISG20	58
	Down	Mitotic spindle organization (GO:0007052)	AURKB;BIRC5;CDC20;SPC24;KIF4A;CENPF;CCNB1;DLGAP5;KIF2C;KNL1	63
		Microtubule cytoskeleton organization involved in mitosis (GO:1902850)	AURKB;BIRC5;CDC20;SPC24;KIF4A;CENPF;CCNB1;DLGAP5;KIF2C;KNL1	56
		Mitotic sister chromatid segregation (GO:0000070)	CCNB1;CEP55;DLGAP5;KIF14;KIF18B;KIF2C;NCAPG;PLK1;CDCA8;CDK1	39
GO Molecular Function 2021	Up	Chemokine activity (GO:0008009)	CXCL10;CXCL11;CCL20;CXCL3;CCL5;CXCL2;CXCL8;CX3CL1;CXCL1;CCL26	18
		DNA binding (GO:0003677)	H1-3;H1-4;H4-16;H2AC4;H2BC5;OASL;EGR4;NFATC2;GRHL1;IRF7;FOSB	141
		Cytokine activity (GO:0005125)	CXCL10;CXCL11;CCL20;CXCL3;CCL5;CXCL2;CXCL8;IFNB1;IL6;CX3CL1	47
	Down	Microtubule binding (GO:0008017)	BIRC5;KIF20A;DLGAP5;KIF14;KIF18B;KIF2C;PLK1;KIFC1;PRC1;GTSE1	62
		Tubulin binding (GO:0015631)	BIRC5;KIF20A;DLGAP5;KIF14;KIF18B;KIF2C;PLK1;STMN1;KIFC1;PRC1	76
		Kinase binding (GO:0019900)	ACTB;ACTA2;AURKB;FOXM1;KIF20A;CCNB1;KIF14;PLK1;CCNA2;CIT	101
KEGG 2021 Human	Up	Viral carcinogenesis	H4C3;H4C2;H4C5;H4C8;H2BC8;H4-16;H2BC5;H4C4;H4C13;H2BC11	56
		NF-kappa B signaling pathway	CXCL3;CXCL2;TNFSF13B;PTGS2;DDX58;GADD45G;MAP3K14;BIRC3	32
		Viral protein interaction with cytokine and cytokine receptor	CXCL10;CXCL11;CCL20;CXCL3;CCL5;CXCL2;CXCL8;IL6;CX3CL1;CXCL1	26
	Down	Cell cycle	CDC20;CCNB1;PLK1;PTTG1;CCNA2;BUB1B;CCNB2;CDC25C;CDK1;BUB1	50
MSigDB Hallmark 2020	Up	Interferon Gamma Response	CXCL10;CXCL11;OASL;RSAD2;MX1;CMPK2;CCL5;IFI44L;IDO1;BATF2	109
		Inflammatory Response	CXCL10;CXCL11;CCL20;CCL5;CXCL8;IFITM1;IL6;IRF7;BST2;CX3CL1	75
		TNF-alpha Signaling via NF-kB	CXCL10;CXCL11;CCL20;CXCL3;EGR3;CCL5;CXCL2;SERPINB2;IFIT2;EGR2	113
	Down	E2F Targets	LMNB1;AURKB;BIRC5;TK1;CDC20;MKI67;RRM2;SPC24;KIF4A;ASF1B	101
		Mitotic Spindle	LMNB1;BIRC5;KIF4A;NEK2;CENPF;ANLN;DLGAP5;KIF2C;PLK1;SHROOM2	67
		G2-M Checkpoint	LMNB1;AURKB;BIRC5;CDC20;MKI67;SLC7A5;KIF4A;NEK2;UBE2C;CENPF	95
Panther 2016	Up	Inflammation mediated by chemokine and cytokine signaling pathway Homo sapiens P00031	CXCL10;CCL20;CCL5;CXCL8;NFATC2;ITGB7;PTGS2;CX3CL1;NFATC1;CCL26	35
	Down	Cytoskeletal regulation by Rho GTPase Homo sapiens P00016	ACTB;ACTA2;ACTG2;STMN1;TUBB;MYLK;MYH11;ACTG1;ARHGAP1;CFL1	25

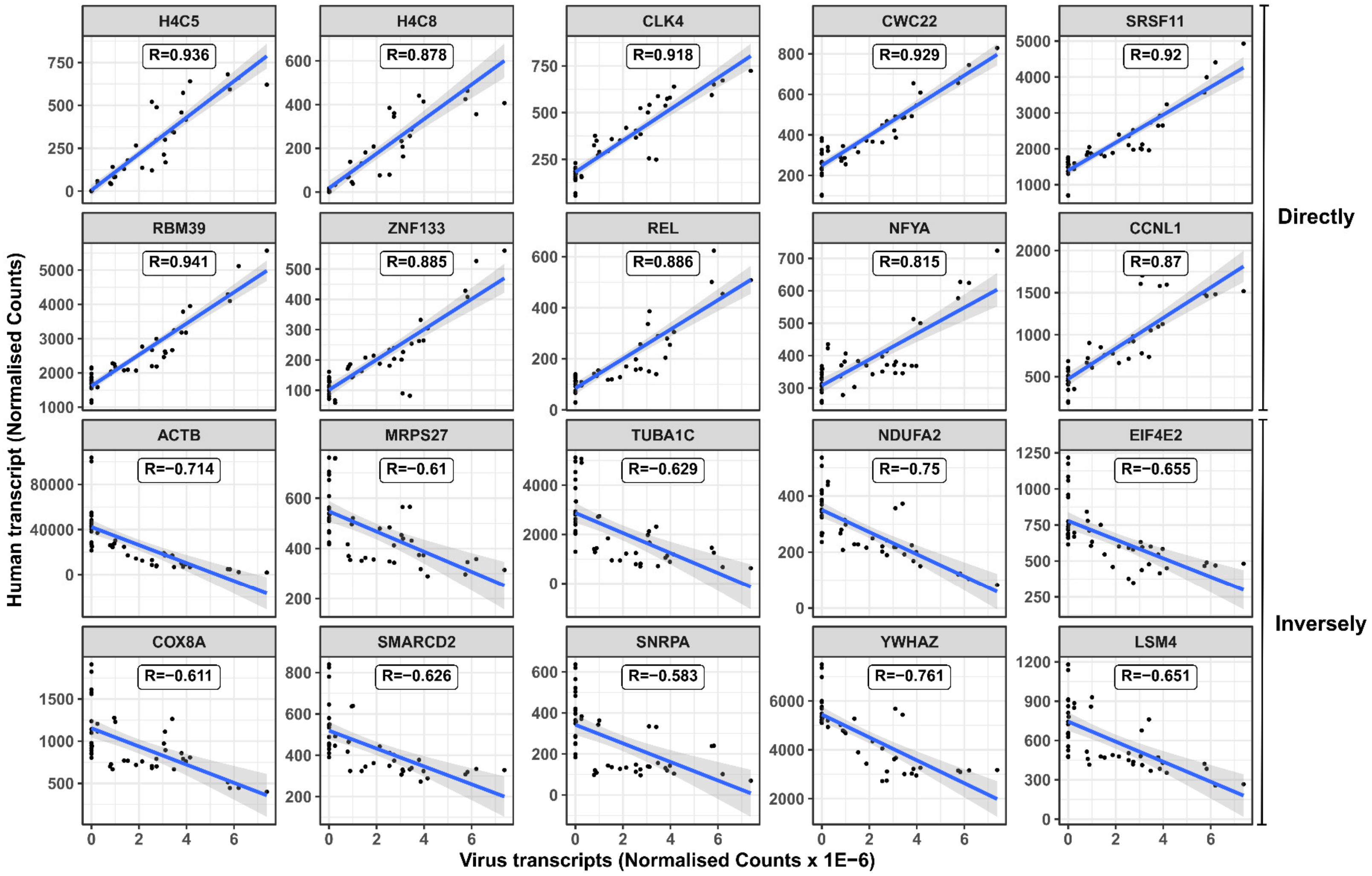

Figure S3. Selected human transcripts that correlate directly or inversely with the total viral RNA in glioma and NB cells infected with *VV-GMCSF-Lact*. Dot plots show correlations between the expression of human transcripts and the normalized count of *VV-GMCSF-Lact* transcripts. R – Pearson's correlation coefficient.

Table S6. Transcription factors involved in regulating genes whose expression correlates with the total number of viral transcripts in glioma and NB cells infected with *VV-GMCSF-Lact*. The table presents the results of Enrichr analysis of the top 300 transcripts showing direct or inverse correlation with the total *VV-GMCSF-Lact* mRNA (Pearson coefficient R > 0.5 for direct, and R < – 0.5 for inverse correlation). Selected Enrichr terms of the "ENCODE and ChEA Consensus TFs from ChIP-X" library are listed.

Correlation with virus transcripts	Term	TF Family	Representative Genes	Number of genes
Direct	ATF2 ENCODE	**ATF2**	ARID4B,CCNL2,DNAJC25,ELMOD3,MAPK8IP3,RBM39,SRSF10,TTC23,ZBTB11,ZSCAN30	73
	BRCA1 ENCODE	**BRCA1**	ABCC5,CCDC142,CCNL1,DDX55,EIF4A2,RBM39,SRSF10,ZBTB11,ZNF133	91
	CREB1 ENCODE<br>CREB1 CHEA	**CREB1**	AFTPH,CCNL1,CCNL2,CWC22,EGR3,POLK,RBM18,REL,RELB,TMEM259,ZBTB11,ZNF451	82
	TAF1 ENCODE	**TAF1**	AKAP8L,CCNL2,CLK1,DDX31,JAK2,MED23,MRPL32,NFKBIA,RBM33,TNFRSF10B,ZNF564	106
	YY1 ENCODE	**YY1**	AFTPH,CCNL1,CCNT2,CLK1,COX19,CWC22,EPC1,RBBP6,RBM39,SRSF10,ZBTB11,ZNF189	96
Inverse	ATF2 ENCODE	**ATF2**	ACAD9,COX8A,EIF4E2,HNRNPC,MRPS27,RAB11B,SLC35A4,SNRPA,TOMM20,TUBA1B,YWHAZ	58
	CREB1 ENCODE	**CREB1**	ACAD9,COPZ1,HNRNPC,JUND,MRPS14,NDUFA7,RAB8A,RNF26,SMARCD2,TBC1D25,TUBA1C,YWHAZ	90
	MAX ENCODE<br>MYC ENCODE	**MYC-MAX**	ACAD9,COX5A,EIF4E2,MRPL12,MRPL15,NFIC,PPIA,SLC35A4,SNRPA,TUBA1B,YWHAE	80
	NFYA ENCODE<br>NFYB ENCODE	**NFY**	ACTB,COPE,COX8A,HNRNPC,JUND,LSM4,MRPS27,RNF20,SLC35A4,TIMM50,TUBA1C,ZDHHC12	122
	TAF1 ENCODE	**TAF1**	ACTB,ACTG1,COPE,EXOSC6,HNRNPC,JUND,MRPL27,NDUFA3,SF3B2,SMARCC1,SNRPC,YWHAZ	111
	YY1 ENCODE	**YY1**	ACO2,COA3,COX5A,EXOC4,HNRNPC,IDH1,MRPL27,MRPS14,NDUFA3,RAB1B,SF3B4	93

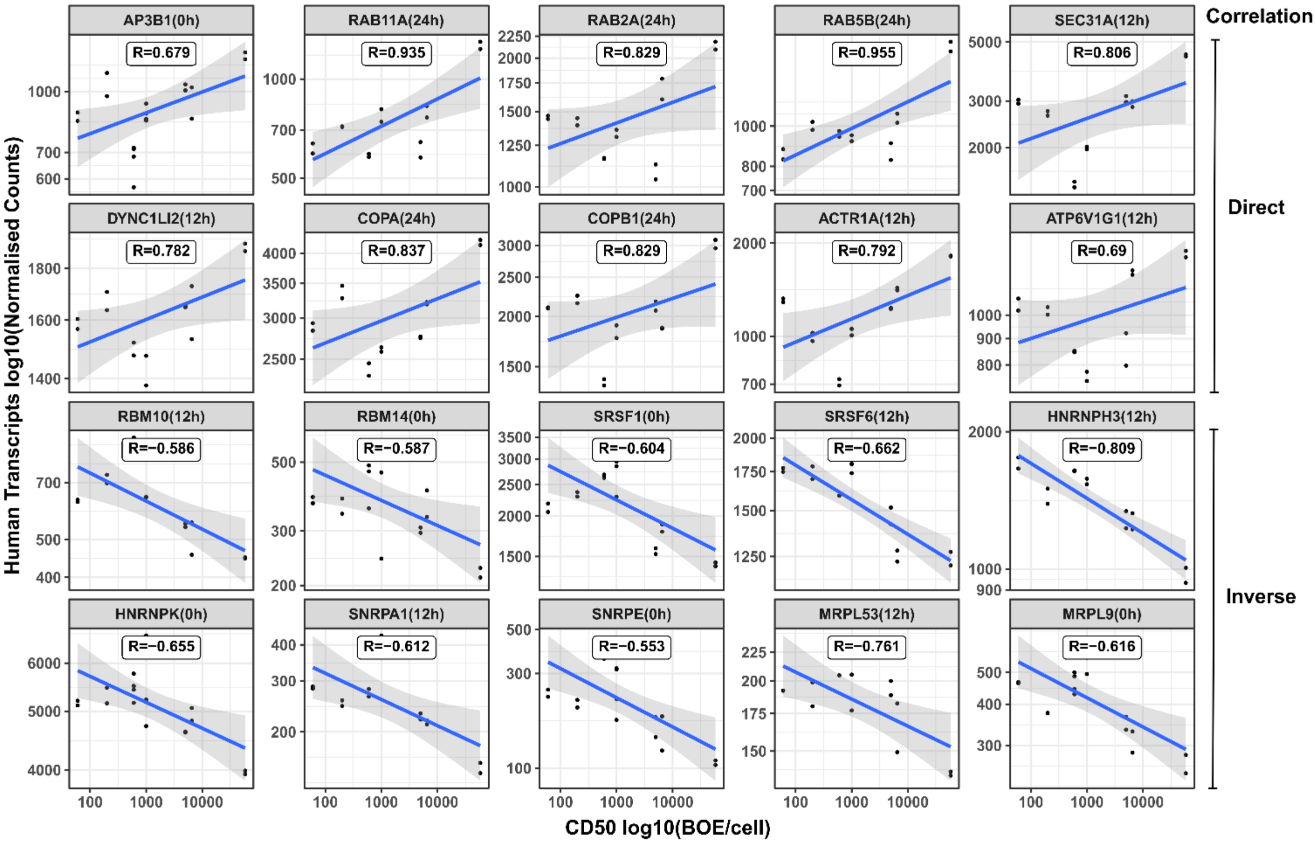

Figure S4. Dot plots of the correlations between the expression of human transcripts and the *VV-GMCSF-Lact* cytotoxicity index (CD_{50}) 0, 12, or 24 h post-infection. Selected human transcripts, which correlate directly or inversely with the cytotoxicity index (CD_{50}) of VV-GMCSF-Lact in glioma and NB cells, are shown. R – Pearson's correlation coefficient.

Table S7. Biological processes and pathways that are associated with gene sets correlating with the total number of viral transcripts in *VV-GMCSF-Lact*-infected human glioma and NB cells. Based on the Enrichr analysis of the top 300 transcripts that correlate directly or inversely with *VV-GMCSF-Lact* mRNA (Pearson coefficient R > 0.5 for direct, and R < – 0.5 for inverse correlation). Selected Enrichr terms from the GO, KEGG, MSigDB Hallmark, and Panther libraries are used.

Enrichr Library	Correlation	Term	Representative Genes	Number of Genes
GO Biological Process 2021	Direct	mRNA processing (GO:0006397)	CCAR1;CWC22;ERCC3;PABPN1;PRMT9;RBM5;SRSF11;U2AF1L4	14
		Negative regulation of type I interferon production (GO:0032480)	HERC5;ITCH;REL;RELB;TBK1	5
		Regulation of RNA splicing (GO:0043484)	CLK1;CLK2;CLK4;CWC22;RBM39;SRSF10;YTHDC1	7
	Inverse	Mitotic spindle organization (GO:0007052)	FLNA;INCENP;KIF3B;KPNB1;MAP4;MAPRE1;NUP43;;RAN;TUBG1	13
		mRNA processing (GO:0006397)	ELAVL1;HNRNPC;LSM3;LSM4;PRCC;RAVER1;SF3B2;SNRPA	12
GO Cellular Component 2021	Direct	Cytoskeleton (GO:0005856)	DGKQ;KLHL2;NEB; RBM39;TTC17;TUBG2;ZC3H12A;ZNF131	12
		H4 histone acetyltransferase complex (GO:1902562)	KANSL1L;MBIP;MSL1	3
		Mitochondrial membrane (GO:0031966)	MRPL32;MTG1;SPG7	3
	Inverse	Cytoskeleton (GO:0005856)	ACTB;ARPC5;FLNA;FSCN1;HNRNPC;KIF3B;TUBA1B	27
		Mitochondrial membrane (GO:0031966)	ATP5F1B;COX5A;MRPL12;MRPS14;NDUFA2;TOMM20;VDAC1	29
GO Molecular Function 2021	Inverse	Nucleosomal DNA binding (GO:0031492)	ACTB;ACTL6A;CHD4;HNRNPC;SMARCA4;SMARCC1;SMARCD2	7
		Ubiquitin protein ligase binding (GO:0031625)	ACTG1;PA2G4;SCAMP3;TPI1;TRIM28;TUBA1B; YWHAE;YWHAZ	15
KEGG 2021 Human	Direct	Herpes simplex virus 1 infection	CHUK;HCFC2;JAK2;NFKBIA;NXF1;TBK1;ZNF133;ZNF189	17
		IL-17 signaling pathway	CCL20;CHUK;CXCL10;CXCL3;NFKBIA;TBK1	6
		TNF signaling pathway	CCL20;CHUK;CXCL10;CXCL3;ITCH;LIF;NFKBIA	7
		Viral carcinogenesis	EGR3;**H2BC4;H2BC5**;NFKBIA;REL;UBR4	11
MSigDB Hallmark 2020	Direct	IL-2/STAT5 Signaling	CXCL10;FAM126B;LIF;MAFF;MXD1;NFKBIZ	6
		p53 Pathway	ABCC5;CDKN2AIP;FBXW7;IP6K2;LIF;MXD1;PIDD1;TSPYL2	8
		TNF-alpha Signaling via NF-kB	CCL20;CCNL1; CXCL3;EGR3;LIF;MAFF;MXD1;REL;RELB;ZC3H12A	11
		Unfolded Protein Response	EIF4A2;NFYA;TSPYL2;ZBTB17	4
	Inverse	G2-M Checkpoint	H2AZ2;HMGB3;KPNA2;KPNB1;LIG3;SMARCC1;STMN1	11
		Myc Targets V1	AIMP2;COX5A; HNRNPC;KPNA2; PSMB2;SMARCC1; VDAC1;	22
		Oxidative Phosphorylation	ACO2;ALDH6A1;ATP5F1B; COX8A;IDH1;NDUFA3; VDAC1	21
		Unfolded Protein Response	BANF1;EIF4EBP1;KHSRP;LSM4;NHP2;YWHAZ	6
Panther 2016	Direct	Apoptosis signaling pathway Homo sapiens P00006	CHUK;HSPA6;NFKBIA;REL;RELB;TNFRSF10B	6
		Inflammation mediated by chemokine and cytokine signaling pathway Homo sapiens P00031	CCL20;CHUK;CXCL10;JAK2;NFATC2;RELB	6
		Toll receptor signaling pathway Homo sapiens P00054	CHUK;NFKBIA;TBK1	3
	Inverse	Cytoskeletal regulation by Rho GTPase Homo sapiens P00016	ACTB;ARPC5;CFL1;DIAPH1;PFN2;RAC1;STMN1;TUBB;TUBB4B	11
		FGF signaling pathway Homo sapiens P00021	PPP2R1A;RAC1;YWHAE;YWHAZ	4
		Integrin signalling pathway Homo sapiens P00034	ACTB;ACTG1;ARPC1A;ARPC5;FLNA;PIK3R2;RAC1	7
--*	Direct	Histone genes*	**H1-3;H1-4;H2AC6;H2BC4;H2BC5;H2BC8;H3C3;H4C2;H4C3;H4C5;H4C8**	11

* – Histone RNAs are presented as a separate group of transcripts independent of Enrichr data that directly correlated with the level of viral RNA, and highlighted in bold.

Table S8. Transcription factors regulating genes whose expression directly or inversely correlates with the *VV-GMCSF-Lact* cytotoxic dose (CD_{50}). Based on the Enrichr analysis of transcripts sets (top 300 according to Pearson R) that directly or inversely correlate with CD_{50} 0, 12, or 24 h post-infection (Pearson coefficient R > 0.5 for direct, and R < –0.5 for inverse correlation). Enrichr terms of the "ENCODE and ChEA Consensus TFs from ChIP-X" library are used.

Correlation with CD_{50}	Term	TF Family	Representative Genes	Number of genes
**Direct** (Resistance)	ATF2 ENCODE	**ATF2**	ACTR3;COPA;EIF4A2;HEXA;RAB11A;RAB1A;SEC24D;TMEM167A;ZNF561	157
	BRCA1 ENCODE	**BRCA1**	AP3B1;ATP6V1H;COPB2;LRRC57;LRRC8A;LZTR1;MYL6;RAB11A;SLC35E1;TMEM127;ZBTB21	176
	CREB1 ENCODE	**CREB1**	ACTR1A;ARF4;EIF4A2;MORF4L1;NR1H2;OXR1;RAB1A;SLC31A1;TMEM165;TUBA1A;ZBTB21	185
	CREB1 CHEA			
	ELF1 ENCODE	**ELF1**	ACTR2;ATP6V1E1;CD46;CDC42;COPB1;GOLGB1;RAB18;SLC25A46;TMED3;YWHAB;ZNF251	149
	TAF1 ENCODE	**TAF1**	ACTR1A;ACTR2;ATP6V1G1;CDC42;COPB1;DDX24;E2F3;EIF4A2;RAB14;SEC31A;SLC10A3;TMEM165	214
	UBTF ENCODE	**UBTF**	ATP6V1G1;E2F3;MAP4K5;MYO1C;NOTCH2;RAB2A;SEC24B;TMEM165;UBE2A;VMP1;ZMYM2	117
	YY1 ENCODE	**YY1**	ATP6AP1;COG5;E2F3;KDM5C;PPP1R12A;RAB14;TMEM135;UBR2;USP15;ZBTB4;ZNF638;ZNFX1	156
**Inverse** (Sensitivity)	BRCA1 ENCODE	**BRCA1**	CCDC124;CNOT6;DHX34;EIF2D;HNRNPA0;JUN;POLR2B;RBM14;RPL14;RPS16;SRSF1;TRA2B;ZNF473	168
	CREB1 ENCODE	**CREB1**	CCDC124;DHX36;EIF4A3;FOSL2;HNRNPD;MRPL24;MRPS15;POLR2B;RBM14;SRSF2;TMEM147	154
	CREB1 CHEA			
	E2F4 ENCODE	**E2F**	ABCB6;CCDC66;CENPM;DHX15;HNRNPAB;MRPL24;MRPS27;NPRL2;NUP155;POLR2D;RBM14;SRSF1	184
	E2F6 ENCODE			
	MAX ENCODE	**MYC-MAX**	CCDC124;DDX11;DHX15;E2F6;FOSL2;HNRNPAB;MRPL24;NUP155;POLR2I;RBM14;SNRNP70;SRSF1	169
	MYC ENCODE			
	NFYA ENCODE	**NFY**	CCDC58;CEP131;HNRNPA0;JUN;METTL17;MRPL11;POLR2I;RBM10;SIN3A;SRSF1;TMEM14C	182
	NFYB ENCODE			
	TAF1 ENCODE	**TAF1**	ADH5;COX4I1;E2F6;HNRNPD;JUN;MRPL9;NDUFAB1;NUP37;PSMA7;RBM10;SRSF2	192
	YY1 ENCODE	**YY1**	COX10;DDX28;E2F6;HNRNPD;MRPL11;MRPL23;NOP58;POLR2D;RBM10;SNRNP40;SRSF2;ZNF764	160

Table S9. Selected biological processes and pathways associated with sets of human genes whose expression correlates with the cytotoxic dose of *VV-GMCSF-Lact* (CD_{50}). Based on the Enrichr analysis of transcripts sets (top 300 according to Pearson R) that directly or inversely correlate with CD_{50} 0, 12, or 24 h of infection (Pearson coefficient R > 0.5 for direct, and R < – 0.5 for inverse correlation). Enrichr terms of the GO, KEGG, MSigDB Hallmark, and Reactome libraries are used.

Enrichr Library	Correlation with CD_{50}	Term	Representative Genes	Number of Genes
GO Biological Process 2021	Direct	Endoplasmic reticulum to Golgi vesicle-mediated transport (GO:0006888)	ARFGAP2;CD59;COPA;DCTN1;DYNC1I2;RAB1A;SEC23A;SPTAN1;TMED9;TRAPPC11	33
		Protein transport (GO:0015031)	AP3B1;ARF1;ATP6AP1;COPB2;LAMP2;MACF1;MYO6;NAPG;PREPL;RAB14;SAR1A	44
	Inverse	mRNA splicing, via spliceosome (GO:0000398)	BCAS2;CPSF7;CSTF2;DHX15;EFTUD2;EIF4A3;FUS;HNRNPA0;ISY1;LSM2;PCBP2;POLR2B	51
		RNA splicing, via transesterification reactions with bulged adenosine as a nucleophile (GO:0000377)	BCAS2;CPSF7;CSTF2;DHX38;EFTUD2;EIF4A3;FUS;HNRNPA0;ISY1;LSM2;PCBP2;POLR2B	51
KEGG 2021 Human	Inverse	Spliceosome	BCAS2;DHX15;EFTUD2;EIF4A3;FUS;HNRNPK;LSM2;MAGOH;PRPF19;RBM25;SF3B4	33
MSigDB Hallmark 2020	Direct	Apoptosis	ADD1;BCAP31;BCL2L2;BIRC2;CAV1;CCND1;CD44;CDKN1A;DAP;FAS;GSN;HSPB1	19
		G2-M Checkpoint	CCND1;E2F3;NOTCH2;PAFAH1B1;PURA	5
		Hypoxia	AK4;ATP7A;CAV1;CDKN1A;CHST3;GBE1;GPI;P4HA2;PDK3;PFKL;PGK1;SDC3;SIAH2	20
		IL-2/STAT5 Signaling	AHNAK;ANXA4;CD44;CKAP4;MYO1C;PHTF2;RNH1;SERPINB6;SYT11;TWSG1;WLS	17
		Mitotic Spindle	BCAR1;CDC42;CLIP1;DST;FLNA;MYO9B;NOTCH2;RALBP1;SPTAN1;SUN2;VCL	21
		mTORC1 Signaling	ACTR2;ACTR3;AK4;CDKN1A;EPRS1;FGL2;GBE1;GPI;GMN;PFKL;PGK1;PSMC6;RAB1A	16
		Oxidative Phosphorylation	ATP6AP1;ATP6V1E1;ATP6V1G1;ATP6V1H;GLUD1;GPI;HADHB;OAT;OGDH	9
		PI3K/AKT/mTOR Signaling	ACTR2;ACTR3;ARF1;CAB39;CDKN1A;CLTC;GRB2;MAPK1;RAC1;TNFRSF1A;YWHAB	11
		Protein Secretion	AP3B1;ARF1;ARFGAP3;ARFIP1;ATP6V1H;ATP7A;CLTC;COPB1;RAB14;SEC24D; VPS4B	22
	Inverse	E2F Targets	CDK4;CENPM;CKS2;HNRNPD;LBR;PAICS;SPC24;SRSF1;SRSF2;SSRP1;TK1;WDR90	16
		G2-M Checkpoint	CDK4;CKS2;CUL3;DKC1;H2AZ1;HNRNPD;HNRNPU;LBR;LIG3;STMN1;TENT4A;TNPO2	19
		Myc Targets V1	CDK4;CSTF2;DHX15;FBL;H2AZ1;HDDC2;HNRNPD;LSM2;PHB2;PSMA7;RACK1;RPL14	27
		Oxidative Phosphorylation	ALDH6A1;ATP5MC2;COX10;FXN;LDHB;MRPL11;MTRF1;SDHC;SLC25A5;UQCR11	24
Reactome 2016	Inverse	mRNA Splicing - Major Pathway Homo sapiens R-HSA-72163	CSTF2;DHX38;HNRNPA0;LSM2;MAGOH;PCBP2;RBMX;SF3B4;SNRNP40;SRSF1;USP39	38
		Processing of Capped Intron-Containing Pre-mRNA Homo sapiens R-HSA-72203	BCAS2;CHTOP;CPSF7;CSTF2;DHX38;EIF4A3;FUS;RBMX;SF3B4;SRSF1;TRA2B;USP39	43

Table S10. A brief review of literature data on properties, targets, and interactions of human transcription factors modulated by *VV-GMCSF-Lact*.

TF	Alias	Description	Ref
ATF2	Activating Transcription Factor 2	Member of the leucine zipper family of DNA binding moonlighting proteins: it forms a homodimer or a heterodimer with c-Jun and stimulates CRE-dependent transcription, also it is a histone acetyltransferase that specifically acetylates the H2B and H4 histones.	[1–4]
BRCA1	Breast and Ovarian Cancer Susceptibility Protein 1	Nuclear phosphoprotein plays a role in maintaining genomic stability, and it also acts as a tumor suppressor, and together with other tumor suppressors forms the multi-subunit protein, BRCA1-associated genome surveillance complex (BASC). BRCA binds to RNA polymerase II and through the C-terminal domain also interacts with histone deacetylase complexes. BRCA1 plays a role in transcription, DNA double-strand break repair, and recombination.	[5–11]
CREB1	Cyclic AMP Response Element (CRE)-Binding Protein Activating Transcription Factor 1	Member of the leucine zipper family of DNA binding proteins. It binds to the cAMP-response element a sequence present in many viral and cellular promoters. Regulates tumor cell proliferation and migration. Involved in different cellular processes including the regulation of GLUT1 expression, thereby mediating glucose transport in cells.	[12–14]
E2F family	E2F1 (E2F Transcription Factor 1)	E2F1 binds the promoter region of genes whose products are involved in cell cycle regulation or DNA replication. The E2F family also plays a crucial role in the control of the action of tumor suppressor proteins and is a target of the transforming proteins of small DNA tumor viruses.	[15–18]
	E2F4 (E2F Transcription Factor 4)	E2F4 plays a critical role in the suppression of proliferation-related genes by binding to all three tumor suppressor proteins, pRB, p107 and p130, but with higher affinity to the last two.	[19–23]
	E2F6 (E2F Transcription Factor 6)	E2F6 lacks the domains that are involved in transactivation and binding to the pRB family members, so is known to be a pRb-independent transcription repressor of E2F-target genes. A critical role of E2F1 and E2F6 was demonstrated in virus-induced malignancies.	[24–27]
CELF1	CUG Triplet Repeat RNA-Binding Protein 1	Member of the CELF/BRUNOL protein family containing two N-terminal RNA recognition motif domains. An RNA-binding protein involved in: an alternative pre-mRNA splicing, translation and mRNA stability. Mediates the inclusion and/or exclusion of an exon in pre-mRNAs that undergo tissue-specific and developmentally regulated alternative splicing. RNA-binding protein CELF1 enhances cell migration, invasion, and chemoresistance by targeting ETS2.	[28–31]
FOSL2	FOS Like 2, AP-1 Transcription Factor Subunit	FOSL2 belongs to FOS family of TFs, also including FOS, FOSB, FOSL1 and encodes leucine zipper proteins, that can dimerize with proteins of the JUN family, thereby forming the transcription factor complex AP-1 in response to extracellular signals (growth factors, hormones, stress, cytokines, inflammation), and binds to specific DNA-binding domains. It was shown that genes whose expression highly correlated with the degree of necrosis in the GMB included transcription factors recently identified as regulators of the mesenchymal transition, including FOSL2.	[32–35]

TF	Alias	Description	Ref
IRF	Interferon Regulatory Factor	IRF proteins have a conserved N-terminal DNA-binding domain with a helix-loop-helix structure and a motif containing five tryptophan residues. They recognize a consensus DNA sequence known as the interferon-stimulated response element (ISRE). In glioma, IRF1, IRF2, IRF5, IRF8, and IRF9 are significantly upregulated compared to normal brain tissue, regardless of infection. It has also been shown that higher mRNA levels of IRF1, IRF2, IRF3, IRF4, IRF5, IRF7, IRF8 and IRF9 correlated with later tumor stages and worse outcomes.	[36–39]
MYC-MAX family	MYC Proto-Oncogene, BHLH Transcription Factor	MYC is a proto-oncogene and encodes a nuclear phosphoprotein that plays a role in cell cycle progression, apoptosis and cellular transformation. The encoded protein forms a heterodimer with the related transcription factor MAX. This complex binds to the E box DNA consensus sequence and regulates the transcription of specific target genes. MYC are aberrantly expressed in GBM and have been linked to the regulation of cell growth and glucose metabolism in GBM. Activation of MYC promotes the Warburg effect (aerobic glycolysis) and induces glycolysis and glutaminolysis. The cells proliferating on a c-*myc*-driven program have lost their ability to process and present antigens by the HLA class I pathway, implying that the nonimmunogenic phenotype of the Burkitt tumor is a direct consequence of the c-*myc* deregulation that is crucial to the process of malignant transformation.	[40–43]
	MAX, MYC-associated protein X	The protein encoded by MAX gene is a member of the basic helix-loop-helix leucine zipper (bHLHZ) family of transcription factors. It is able to form homodimers and heterodimers with other family members, which include Mad, Mxi1 and Myc. Myc is an oncoprotein implicated in cell proliferation, differentiation and apoptosis. The glioma cells displayed alternative splicing patterns of MAX mRNAs encoding Max proteins which either suppress (Max) or augment (delta Max) the transforming activity of MYC. Glioma cells may coexpress several MYC genes, thus resembling fetal neural cells and alternative processing of MAX mRNA in some glioma cells results in delta MAX encoding mRNAs not seen in normal fetal brain.	[44–46]
NFY family	Nuclear Transcription Factor Y Subunit Alpha	Nuclear transcription factor Y (NFY) consists of three different subunits NFYA, NFYB and NFYC, which form the heterotrimeric complex. NFY can function as both an activator and a repressor, depending on its interacting cofactors. NFYA positively regulates the transcription of the core component BMAL1. Previously, studies on the effect of TF expression on the life cycle of viruses showed that an increase in NFY expression led to the switch of EBV from a latent state to a lytic one.	[47–51].
	Nuclear Transcription Factor Y Subunit Beta	NFYC and NFYB form a heterodimer which subsequently binds NFYA, promoting its CCAAT box-binding capability on several target genes. The complex has demonstrated the ability to regulate the transcription of several genes including some involved in cell cycle regulation and cellular differentiation. NFYC has been shown to be variably expressed in human glioma samples and may play a role in glioma cell proliferation and development through cell cycle regulation and cell adhesion molecules.	[52–54]

TF	Alias	Description	Ref
RELA	RELA Proto-Oncogene, NF-kB Subunit	RELA, a member of the NF-κB family (p65, p50, p52, c-Rel, and RelB), was known to interact with the inhibitor of κB (IκB) proteins in the cytoplasm. Besides its activity as a direct transcriptional activator, it is also able to modulate promoters accessibility to transcription factors and thereby indirectly regulate gene expression. Abnormal RELA expression is linked to improper development, inflammatory diseases, and cancer. Decreased A20 expression prevents the growth and survival of GBM stem cells by reducing RELA phosphorylation and inhibiting cell cycle progression. The VACV K1 protein inhibits RelA acetylation and RelA-CBP interactions, suggesting that K1 performs its inhibitory function in the nucleus. VACV encodes for several NF-kB inhibitors. Most of these proteins act prior to nuclear translocation of NF-κB. Thus, K1 is the first VACV protein to act in the nucleus to prevent NF-κB activation. K1 is unique also because the other known NF-κB inhibitory proteins expressed by VACV do not possess ARDs.	[55–59]
REST	RE1 Silencing Transcription Factor	The REST transcription factor, also known as NRSF (neuron-restrictive silencer factor), inhibits the binding of PRC1 and PRC2 to sites near the promoter and, by binding to the CBX subunit, promotes H3K27me3 label-independent landing of PRC1 to sites remote from the promoter. It restricts neuronal gene expression by binding to the neuron-restrictive silencer element (NRSE/RE1) domain in neuron-specific genes. REST represses the DRD2 gene, and it is suggested that the REST-DRD2 mechanism can be used to stratify GBM into separate subtypes: HRLD and LRHD. REST overexpression has been found to positively regulate the oncogenic properties of many medulloblastoma, GBM, and neuroblastoma models. In contrast, deletion of REST and p53 resulted in tumors similar to the proneural GBM subtype in adult mice, suggesting that REST function in GBM tumors is context-dependent. In the case of REST, while its oncogenic function depends on its role in regulating properties such as maintaining stemness, cell invasion, and apoptosis, its tumor suppressor function depends on its role in maintaining genome integrity.	[60–62]
SRF	Serum Response Factor	SRF is a ubiquitous nuclear protein important for cell proliferation, differentiation, the cell cycle, apoptosis, the function of which is required to regulate the transcription of numerous growth factor-induced genes such as the c-fos oncogene, and muscle-specific actin genes. It is a member of the MADS eukaryotic transcription factor superfamily (MCM1, Agamous, Deficiens and SRF). Related to testis-specific and pathogenesis protein 1 (RTVP-1) is highly expressed in gliomas and plays a role in the migration of these cells. SRF regulated the migration of glioma cells and its effect was partially mediated by RTVP-1. It was shown that role of the HCV core protein in promoting metastasis is at least partially dependent on the activation of the MAPK/ERK/PEA3-SRF/c-Fos/MMP2/MMP9 axis	[63–66]
TAF1	TATA-Box Binding Protein Associated Factor 1	TAF1 is the largest subunit of the polymerase II PIC, and acts as a transcriptional coactivator. This protein is involved in the recognition of promoters and the modification of general transcription factors. TAF1 is altered in 3.85% of glioblastoma patients. Elevated FOXD2-AS1 lncRNA levels were	[67–72]

TF	Alias	Description	Ref
		found to activate NOTCH1 by recruiting TAF-1 to the NOTCH1 promoter region, thereby promoting stemness and proliferation of GSCs, and suppressing GSC differentiation and apoptosis. TAF1 participates in HCV viral transcription initiation and elongation. TAF1 is one of the most commonly mutated genes in hepatitis B virus-related HCCs and certain associations existed between MRI features and high-frequency mutations.	
UBTF	Nucleolar Transcription Factor 1	This gene encodes a member of the HMG-box family of DNA-binding proteins. The protein plays a critical role in ribosomal RNA transcription as a component of the pre-initiation complex, mediating the recruitment of RNA polymerase I to rDNA promoter regions. UBTF is involved in carcinogenesis and progression of a few cancers. It was shown an antiviral impact of UBTF on HSV-1 replication in HeLa cells.	[73,74]
YY1	YY1 Transcription Factor	Yin Yang-1 (YY1) is a common transcription factor belonging to the GLI-Kruppel family of zinc finger DNA-binding proteins. This TF is involved in the repression and activation of various promoters depending on interacting partners, promoter context and chromatin structure. YY1 can direct histone deacetylases and histone acetyltransferases to the promoter to activate or repress it, thus involving histone modification into YY1 function. YY1 is involved in the regulation of tumor cell growth pathways, epithelial-mesenchymal transition, invasion, and resistance to chemotherapy. YY1 was shown to be highly expressed in GBM tissues and cells. YY1 can promote GSC self-renewal by enhancing SUMOylase SENP1 transcription and enhancing METTL3 methylase activity, resulting in an increase in the m6A mRNA modification level of MYC. Approximately half of the intermediate and late gene transcriptional promoters of the vaccinia virus have a binding site for the cellular transcription factor YY1 that overlaps the initiator elements. YY1 negatively regulates vaccinia promoters by binding to their initiator elements.	[75–78]

References

1. Yu, T.; Li, Y.J.; Bian, A.H.; Zuo, H. Bin; Zhu, T.W.; Ji, S.X.; Kong, F.; Yin, D.Q.; Wang, C.B.; Wang, Z.F.; et al. The Regulatory Role of Activating Transcription Factor 2 in Inflammation. *Mediators Inflamm.* **2014**, *2014*, 1–10, doi:10.1155/2014/950472.
2. Kawasaki, H.; Schiltz, L.; Chiu, R.; Itakura, K.; Taira, K.; Nakatani, Y.; Yokoyama, K.K. ATF-2 Has Intrinsic Histone Acetyltransferase Activity Which Is Modulated by Phosphorylation. *Nature* **2000**, *405*, 195–200, doi:10.1038/35012097.
3. Jiang, F.-F.; Wang, R.-Q.; Guo, C.-Y.; Zheng, K.; Liu, H.-L.; Su, L.; Xie, S.-S.; Chen, H.-C.; Liu, Z.-F. Phospho-Proteomics Identifies a Critical Role of ATF2 in Pseudorabies Virus Replication. *Virol. Sin.* **2022**, *37*, 591–600, doi:10.1016/j.virs.2022.06.003.
4. Turton, K.L.; Meier-Stephenson, V.; Badmalia, M.D.; Coffin, C.S.; Patel, T.R. Host Transcription Factors in Hepatitis B Virus RNA Synthesis. *Viruses* **2020**, *12*, 160, doi:10.3390/v12020160.
5. Fong, P.C.; Boss, D.S.; Yap, T.A.; Tutt, A.; Wu, P.; Mergui-Roelvink, M.; Mortimer, P.; Swaisland, H.; Lau, A.; O'Connor, M.J.; et al. Inhibition of Poly(ADP-Ribose) Polymerase in Tumors from BRCA Mutation Carriers. *N. Engl. J. Med.* **2009**, *361*, 123–134, doi:10.1056/NEJMoa0900212.
6. Thakar, T.; Dhoonmoon, A.; Straka, J.; Schleicher, E.M.; Nicolae, C.M.; Moldovan, G.-L. Lagging Strand Gap Suppression Connects BRCA-Mediated Fork Protection to Nucleosome Assembly through PCNA-Dependent CAF-1 Recycling. *Nat. Commun.* **2022**, *13*, 5323, doi:10.1038/s41467-022-33028-y.
7. Wang, Y.; Cortez, D.; Yazdi, P.; Neff, N.; Elledge, S.J.; Qin, J. BASC, a Super Complex of BRCA1-Associated Proteins Involved in the Recognition and Repair of Aberrant DNA Structures. *Genes Dev.* **2000**, *14*, 927–939, doi:10.1101/gad.14.8.927.
8. Maul, G.G.; Jensen, D.E.; Ishov, A.M.; Herlyn, M.; Rauscher, F.J. Nuclear Redistribution of BRCA1 during Viral Infection. *Cell Growth Differ.* **1998**, *9*.
9. Iqbal, J.; Ansari, M.A.; Kumar, B.; Dutta, D.; Roy, A.; Chikoti, L.; Pisano, G.; Dutta, S.; Vahedi, S.; Veettil, M.V.; et al. Histone H2B-IFI16 Recognition of Nuclear Herpesviral Genome Induces Cytoplasmic Interferon-β Responses. *PLOS Pathog.* **2016**, *12*, e1005967, doi:10.1371/journal.ppat.1005967.
10. Hiraike, H.; Wada-Hiraike, O.; Nakagawa, S.; Koyama, S.; Miyamoto, Y.; Sone, K.; Tanikawa, M.; Tsuruga, T.; Nagasaka, K.; Matsumoto, Y.; et al. Identification of DBC1 as a Transcriptional Repressor for BRCA1. *Br. J. Cancer* **2010**, *102*, 1061–1067, doi:10.1038/sj.bjc.6605577.
11. Lin, K.; Baritaki, S.; Vivarelli, S.; Falzone, L.; Scalisi, A.; Libra, M.; Bonavida, B. The Breast Cancer Protooncogenes HER2, BRCA1 and BRCA2 and Their Regulation by the INOS/NOS2 Axis. *Antioxidants* 2022.
12. Zhu, L.; Gao, T.; Huang, Y.; Jin, J.; Wang, D.; Zhang, L.; Jin, Y.; Li, P.; Hu, Y.; Wu, Y.; et al. Ebola Virus VP35 Hijacks the PKA-CREB1 Pathway for Replication and Pathogenesis by AKIP1 Association. *Nat. Commun.* **2022**, *13*, 2256, doi:10.1038/s41467-022-29948-4.
13. Li, Y.; Fu, Y.; Hu, X.; Sun, L.; Tang, D.; Li, N.; Peng, F.; Fan, X. The HBx–CTTN Interaction Promotes Cell Proliferation and Migration of Hepatocellular Carcinoma via CREB1. *Cell Death Dis.* **2019**, *10*, 405, doi:10.1038/s41419-019-1650-x.
14. Chen, J.; Zhang, C.; Mi, Y.; Chen, F.; Du, D. CREB1 Regulates Glucose Transport of Glioma Cell Line U87 by Targeting GLUT1. *Mol. Cell. Biochem.* **2017**, *436*, 79–86, doi:10.1007/s11010-017-3080-3.
15. Tanimoto, T.; Tazawa, H.; Ieda, T.; Nouso, H.; Tani, M.; Oyama, T.; Urata, Y.; Kagawa, S.; Noda, T.;

Fujiwara, T. Elimination of MYCN-Amplified Neuroblastoma Cells by Telomerase-Targeted Oncolytic Virus via MYCN Suppression. *Mol. Ther. - Oncolytics* **2020**, *18*, 14–23, doi:10.1016/j.omto.2020.05.015.

16. Sharon, E.; Volchek, L.; Frenkel, N. Human Herpesvirus 6 (HHV-6) Alters E2F1/Rb Pathways and Utilizes the E2F1 Transcription Factor to Express Viral Genes. *Proc. Natl. Acad. Sci.* **2014**, *111*, 451–456, doi:10.1073/pnas.1308854110.
17. Workman, A.; Jones, C. Analysis of the Cell Cycle Regulatory Protein (E2F1) after Infection of Cultured Cells with Bovine Herpesvirus 1 (BHV-1) or Herpes Simplex Virus Type 1 (HSV-1). *Virus Res.* **2011**, *160*, 66–73, doi:10.1016/j.virusres.2011.05.009.
18. Kundu, M.; Srinivasan, A.; Pomerantz, R.J.; Khalili, K. Evidence That a Cell Cycle Regulator, E2F1, down-Regulates Transcriptional Activity of the Human Immunodeficiency Virus Type 1 Promoter. *J. Virol.* **1995**, *69*, 6940–6946, doi:10.1128/jvi.69.11.6940-6946.1995.
19. DuPree, E.L.; Mazumder, S.; Almasan, A. Genotoxic Stress Induces Expression of E2F4, Leading to Its Association with P130 in Prostate Carcinoma Cells. *Cancer Res.* **2004**, *64*, 4390–4393, doi:10.1158/0008-5472.CAN-03-3695.
20. Sáenz-Robles, M.T.; Markovics, J.A.; Chong, J.-L.; Opavsky, R.; Whitehead, R.H.; Leone, G.; Pipas, J.M. Intestinal Hyperplasia Induced by Simian Virus 40 Large Tumor Antigen Requires E2F2. *J. Virol.* **2007**, *81*, 13191–13199, doi:10.1128/JVI.01658-07.
21. Allmann, S.; Mayer, L.; Olma, J.; Kaina, B.; Hofmann, T.G.; Tomicic, M.T.; Christmann, M. Benzo[a]Pyrene Represses DNA Repair through Altered E2F1/E2F4 Function Marking an Early Event in DNA Damage-Induced Cellular Senescence. *Nucleic Acids Res.* **2020**, *48*, 12085–12101, doi:10.1093/nar/gkaa965.
22. Wei, J.; Shi, Y.; Zou, C.; Zhang, H.; Peng, H.; Wang, S.; Xia, L.; Yang, Y.; Zhang, X.; Liu, J.; et al. Cellular Id1 Inhibits Hepatitis B Virus Transcription by Interacting with the Novel Covalently Closed Circular DNA-Binding Protein E2F4. *Int. J. Biol. Sci.* **2022**, *18*, 65–81, doi:10.7150/ijbs.62106.
23. Olgiate, J.; Ehmann, G.L.; Vidyarthi, S.; Hilton, M.J.; Bachenheimer, S.L. Herpes Simplex Virus Induces Intracellular Redistribution of E2F4 and Accumulation of E2F Pocket Protein Complexes. *Virology* **1999**, *258*, 257–270, doi:10.1006/viro.1999.9755.
24. Pei, Y.; Banerjee, S.; Sun, Z.; Jha, H.C.; Saha, A.; Robertson, E.S. EBV Nuclear Antigen 3C Mediates Regulation of E2F6 to Inhibit E2F1 Transcription and Promote Cell Proliferation. *PLoS Pathog.* **2016**, *12*, doi:10.1371/journal.ppat.1005844.
25. McLaughlin-Drubin, M.E.; Huh, K.-W.; Mu¨nger, K. Human Papillomavirus Type 16 E7 Oncoprotein Associates with E2F6. *J. Virol.* **2008**, *82*, 8695–8705, doi:10.1128/JVI.00579-08.
26. McLaughlin-Drubin, M.E.; Crum, C.P.; Münger, K. Human Papillomavirus E7 Oncoprotein Induces KDM6A and KDM6B Histone Demethylase Expression and Causes Epigenetic Reprogramming. *Proc. Natl. Acad. Sci.* **2011**, *108*, 2130–2135, doi:10.1073/pnas.1009933108.
27. Xu, X.; Bieda, M.; Jin, V.X.; Rabinovich, A.; Oberley, M.J.; Green, R.; Farnham, P.J. A Comprehensive ChIP-Chip Analysis of E2F1, E2F4, and E2F6 in Normal and Tumor Cells Reveals Interchangeable Roles of E2F Family Members. *Genome Res.* **2007**, *17*, doi:10.1101/gr.6783507.
28. Wang, H.; Huang, R.; Guo, W.; Qin, X.; Yang, Z.; Yuan, Z.; Wei, Y.; Mo, C.; Zeng, Z.; Luo, J.; et al. RNA-Binding Protein CELF1 Enhances Cell Migration, Invasion, and Chemoresistance by Targeting ETS2 in Colorectal Cancer. *Clin. Sci.* **2020**, *134*, doi:10.1042/CS20191174.
29. Lin, P.-C.; Huang, H.-D.; Chang, C.-C.; Chang, Y.-S.; Yen, J.-C.; Lee, C.-C.; Chang, W.-H.; Liu, T.-C.;

Chang, J.-G. Long Noncoding RNA TUG1 Is Downregulated in Non-Small Cell Lung Cancer and Can Regulate CELF1 on Binding to PRC2. *BMC Cancer* **2016**, *16*, 583, doi:10.1186/s12885-016-2569-6.

30. Xia, L.; Sun, C.; Li, Q.; Feng, F.; Qiao, E.; Jiang, L.; Wu, B.; Ge, M. CELF1 Is Up-Regulated in Glioma and Promotes Glioma Cell Proliferation by Suppression of CDKN1B. *Int. J. Biol. Sci.* **2015**, *11*, 1314–1324, doi:10.7150/ijbs.11344.
31. Siddam, A.D.; Gautier-Courteille, C.; Perez-Campos, L.; Anand, D.; Kakrana, A.; Dang, C.A.; Legagneux, V.; Méreau, A.; Viet, J.; Gross, J.M.; et al. The RNA-Binding Protein Celf1 Post-Transcriptionally Regulates P27Kip1 and Dnase2b to Control Fiber Cell Nuclear Degradation in Lens Development. *PLOS Genet.* **2018**, *14*, e1007278, doi:10.1371/journal.pgen.1007278.
32. Wan, X.; Guan, S.; Hou, Y.; Qin, Y.; Zeng, H.; Yang, L.; Qiao, Y.; Liu, S.; Li, Q.; Jin, T.; et al. FOSL2 Promotes VEGF-Independent Angiogenesis by Transcriptionnally Activating Wnt5a in Breast Cancer-Associated Fibroblasts. *Theranostics* **2021**, *11*, 4975–4991, doi:10.7150/thno.55074.
33. Cooper, L.A.D.; Gutman, D.A.; Chisolm, C.; Appin, C.; Kong, J.; Rong, Y.; Kurc, T.; Van Meir, E.G.; Saltz, J.H.; Moreno, C.S.; et al. The Tumor Microenvironment Strongly Impacts Master Transcriptional Regulators and Gene Expression Class of Glioblastoma. *Am. J. Pathol.* **2012**, doi:10.1016/j.ajpath.2012.01.040.
34. Gazon, H.; Barbeau, B.; Mesnard, J.M.; Peloponese, J.M. Hijacking of the AP-1 Signaling Pathway during Development of ATL. *Front. Microbiol.* **2018**, *8*, 1–13, doi:10.3389/fmicb.2017.02686.
35. Ye, N.; Ding, Y.; Wild, C.; Shen, Q.; Zhou, J. Small Molecule Inhibitors Targeting Activator Protein 1 (AP-1). *J. Med. Chem.* 2014.
36. Murtas, D.; Maric, D.; De Giorgi, V.; Reinboth, J.; Worschech, A.; Fetsch, P.; Filie, A.; Ascierto, M.L.; Bedognetti, D.; Liu, Q.; et al. IRF-1 Responsiveness to IFN-γ Predicts Different Cancer Immune Phenotypes. *Br. J. Cancer* **2013**, *109*, 76–82, doi:10.1038/bjc.2013.335.
37. Yanai, H.; Negishi, H.; Taniguchi, T. The IRF Family of Transcription Factors: Inception, Impact and Implications in Oncogenesis. *Oncoimmunology* **2012**, *1*, 1376–1386, doi:10.4161/onci.22475.
38. Lei, J.; Zhou, M.-H.; Zhang, F.-C.; Wu, K.; Liu, S.-W.; Niu, H.-Q. Interferon Regulatory Factor Transcript Levels Correlate with Clinical Outcomes in Human Glioma. *Aging (Albany. NY).* **2021**, *13*, 12086–12098, doi:10.18632/aging.202915.
39. Dhungel, P.; Cantu, F.M.; Molina, J.A.; Yang, Z. Vaccinia Virus as a Master of Host Shutoff Induction: Targeting Processes of the Central Dogma and Beyond. *Pathogens* **2020**, doi:10.3390/pathogens9050400.
40. Dhanasekaran, R.; Deutzmann, A.; Mahauad-Fernandez, W.D.; Hansen, A.S.; Gouw, A.M.; Felsher, D.W. The MYC Oncogene — the Grand Orchestrator of Cancer Growth and Immune Evasion. *Nat. Rev. Clin. Oncol.* **2022**, *19*, 23–36, doi:10.1038/s41571-021-00549-2.
41. Patange, S.; Ball, D.A.; Wan, Y.; Karpova, T.S.; Girvan, M.; Levens, D.; Larson, D.R. MYC Amplifies Gene Expression through Global Changes in Transcription Factor Dynamics. *Cell Rep.* **2022**, *38*, 110292, doi:10.1016/j.celrep.2021.110292.
42. Wang, G.; Wang, J.; Zhao, H.; Wang, J.; Tony To, S.S. The Role of Myc and Let-7a in Glioblastoma, Glucose Metabolism and Response to Therapy. *Arch. Biochem. Biophys.* **2015**, *580*, 84–92, doi:10.1016/j.abb.2015.07.005.
43. Staege, M.S.; Lee, S.P.; Frisan, T.; Mautner, J.; Scholz, S.; Pajic, A.; Rickinson, A.B.; Masucci, M.G.; Polack, A.; Bornkamm, G.W. MYC Overexpression Imposes a Nonimmunogenic Phenotype on Epstein-Barr Virus-Infected B Cells. *Proc. Natl. Acad. Sci. U. S. A.* **2002**, doi:10.1073/pnas.072495599.

44. Cascón, A.; Robledo, M. MAX and MYC: A Heritable Breakup. *Cancer Res.* **2012**, *72*, 3119–3124, doi:10.1158/0008-5472.CAN-11-3891.
45. Hurlin, P.J.; Huang, J. The MAX-Interacting Transcription Factor Network. *Semin. Cancer Biol.* **2006**, *16*, 265–274, doi:10.1016/j.semcancer.2006.07.009.
46. Hirvonen, H.; Salonen, R.; Sandberg, M.; Vuorio, E.; Västrik, I.; Kotilainen, E.; Kalimo, H. Differential Expression of Myc, Max and RB1 Genes in Human Gliomas and Glioma Cell Lines. *Br. J. Cancer* **1994**, *69*, 16–25, doi:10.1038/bjc.1994.3.
47. Tsujimoto, G.; Ito, R.; Yoshikawa, K.; Ueki, C.; Okada, N. NFYA Promotes the Anti-Tumor Effects of Gluconeogenesis in Hepatocellular Carcinoma through the Regulation of PCK1 Expression. *Front. Cell Dev. Biol.* **2022**, *10*, doi:10.3389/fcell.2022.983599.
48. Li, Y.; Xiao, X.; Chen, H.X.; Chen, Z.; Hu, K.S.; Yin, D. Transcription Factor NFYA Promotes G1/S Cell Cycle Transition and Cell Proliferation by Transactivating Cyclin D1 and CDK4 in Clear Cell Renal Cell Carcinoma. *Am. J. Cancer Res.* **2020**.
49. Chia, M.C.; Leung, A.; Krushel, T.; Alajez, N.M.; Lo, K.W.; Busson, P.; Klamut, H.J.; Bastianutto, C.; Liu, F.-F. Nuclear Factor-Y and Epstein Barr Virus in Nasopharyngeal Cancer. *Clin. Cancer Res.* **2008**, *14*, 984–994, doi:10.1158/1078-0432.CCR-07-0828.
50. Tan, X.; Zhang, H.; Yang, Z.; Wei, Z.; Li, Y.; Chen, J.; Sun, Z. NF-YA Transcription Factors Suppress Jasmonic Acid-Mediated Antiviral Defense and Facilitate Viral Infection in Rice. *PLoS Pathog.* **2022**, *18*, e1010548, doi:10.1371/journal.ppat.1010548.
51. Court, F.; Le Boiteux, E.; Fogli, A.; Müller-Barthélémy, M.; Vaurs-Barrière, C.; Chautard, E.; Pereira, B.; Biau, J.; Kemeny, J.-L.; Khalil, T.; et al. Transcriptional Alterations in Glioma Result Primarily from DNA Methylation-Independent Mechanisms. *Genome Res.* **2019**, *29*, 1605–1621, doi:10.1101/gr.249219.119.
52. Tharyan, R.G.; Annibal, A.; Schiffer, I.; Laboy, R.; Atanassov, I.; Weber, A.L.; Gerisch, B.; Antebi, A. NFYB-1 Regulates Mitochondrial Function and Longevity via Lysosomal Prosaposin. *Nat. Metab.* **2020**, *2*, 387–396, doi:10.1038/s42255-020-0200-2.
53. Lu, T.; Li, C.; Xiang, C.; Gong, Y.; Peng, W.; Hou, F.; Chen, C. Over-Expression of NFYB Affects Stromal Cells Reprogramming and Predicts Worse Survival in Gastric Cancer Patients. *Aging (Albany. NY).* **2022**, *14*, 7851–7865, doi:10.18632/aging.204294.
54. Cui, H.; Zhang, M.; Wang, Y.; Wang, Y. NF-YC in Glioma Cell Proliferation and Tumor Growth and Its Role as an Independent Predictor of Patient Survival. *Neurosci. Lett.* **2016**, *631*, 40–49, doi:10.1016/j.neulet.2016.08.003.
55. Kim, M.-Y.; Koh, D.-I.; Choi, W.-I.; Jeon, B.-N.; Jeong, D.; Kim, K.-S.; Kim, K.; Kim, S.-H.; Hur, M.-W. ZBTB2 Increases PDK4 Expression by Transcriptional Repression of RelA/P65. *Nucleic Acids Res.* **2015**, *43*, 1609–1625, doi:10.1093/nar/gkv026.
56. Yang, N.; Wang, P.; Wang, W.J.; Song, Y.Z.; Liang, Z.Q. Inhibition of Cathepsin L Sensitizes Human Glioma Cells to Ionizing Radiation in Vitro through NF-KB Signaling Pathway. *Acta Pharmacol. Sin.* **2015**, doi:10.1038/aps.2014.148.
57. Hjelmeland, A.B.; Wu, Q.; Wickman, S.; Eyler, C.; Heddleston, J.; Shi, Q.; Lathia, J.D.; MacSwords, J.; Lee, J.; McLendon, R.E.; et al. Targeting A20 Decreases Glioma Stem Cell Survival and Tumor Growth. *PLoS Biol.* **2010**, *8*, e1000319, doi:10.1371/journal.pbio.1000319.
58. Han, N.; Yang, L.; Zhang, X.; Zhou, Y.; Chen, R.; Yu, Y.; Dong, Z.; Zhang, M. LncRNA MATN1-AS1 Prevents Glioblastoma Cell from Proliferation and Invasion via RELA Regulation and MAPK Signaling

Pathway. *Ann. Transl. Med.* **2019**, *7*, 784–784, doi:10.21037/atm.2019.11.36.

59. Bravo Cruz, A.G.; Shisler, J.L. Vaccinia Virus K1 Ankyrin Repeat Protein Inhibits NF-KB Activation by Preventing RelA Acetylation. *J. Gen. Virol.* **2016**, *97*, 2691–2702, doi:10.1099/jgv.0.000576.

60. Zhao, Y.; Zhu, M.; Yu, Y.; Qiu, L.; Zhang, Y.; He, L.; Zhang, J. Brain REST/NRSF Is Not Only a Silent Repressor but Also an Active Protector. *Mol. Neurobiol.* **2017**, *54*, 541–550, doi:10.1007/s12035-015-9658-4.

61. Marisetty, A.L.; Lu, L.; Veo, B.L.; Liu, B.; Coarfa, C.; Kamal, M.M.; Kassem, D.H.; Irshad, K.; Lu, Y.; Gumin, J.; et al. REST-DRD2 Mechanism Impacts Glioblastoma Stem Cell-Mediated Tumorigenesis. *Neuro. Oncol.* **2019**, *21*, 775–785, doi:10.1093/neuonc/noz030.

62. Fuller, G.N.; Su, X.; Price, R.E.; Cohen, Z.R.; Lang, F.F.; Sawaya, R.; Majumder, S. Many Human Medulloblastoma Tumors Overexpress Repressor Element-1 Silencing Transcription (REST)/Neuron-Restrictive Silencer Factor, Which Can Be Functionally Countered by REST-VP16. *Mol. Cancer Ther.* **2005**, doi:10.1158/1535-7163.mct-04-0228.

63. Schratt, G.; Weinhold, B.; Lundberg, A.S.; Schuck, S.; Berger, J.; Schwarz, H.; Weinberg, R.A.; Ru¨ther, U.; Nordheim, A. Serum Response Factor Is Required for Immediate-Early Gene Activation yet Is Dispensable for Proliferation of Embryonic Stem Cells. *Mol. Cell. Biol.* **2001**, *21*, 2933–2943, doi:10.1128/MCB.21.8.2933-2943.2001.

64. Shore, P.; Sharrocks, A.D. The MADS-Box Family of Transcription Factors. *Eur. J. Biochem.* **1995**, *229*, 1–13, doi:10.1111/j.1432-1033.1995.tb20430.x.

65. Ziv-Av, A.; Taller, D.; Attia, M.; Xiang, C.; Lee, H.K.; Cazacu, S.; Finniss, S.; Kazimirsky, G.; Sarid, R.; Brodie, C. RTVP-1 Expression Is Regulated by SRF Downstream of Protein Kinase C and Contributes to the Effect of SRF on Glioma Cell Migration. *Cell. Signal.* **2011**, doi:10.1016/j.cellsig.2011.07.001.

66. Zhao, L.; Sun, X.; Chen, L.; Feng, X.; Yang, X.; Zou, P.; Wang, X.; Zhang, R. Hepatitis C Virus Core Protein Promotes the Metastasis of Human Hepatocytes by Activating the MAPK/ERK/PEA3-SRF/c-Fos/MMPs Axis. *Arch. Med. Res.* **2022**, *53*, 469–482, doi:10.1016/j.arcmed.2022.06.004.

67. Xu, Y.; Man, N.; Karl, D.; Martinez, C.; Liu, F.; Sun, J.; Martinez, C.J.; Martin, G.M.; Beckedorff, F.; Lai, F.; et al. TAF1 Plays a Critical Role in AML1-ETO Driven Leukemogenesis. *Nat. Commun.* **2019**, *10*, 4925, doi:10.1038/s41467-019-12735-z.

68. Wang, Y.; Cheng, Y.; Yang, Q.; Kuang, L.; Liu, G. Overexpression of FOXD2-AS1 Enhances Proliferation and Impairs Differentiation of Glioma Stem Cells by Activating the NOTCH Pathway via TAF-1. *J. Cell. Mol. Med.* **2022**, *26*, 2620–2632, doi:10.1111/jcmm.17268.

69. Tavassoli, P.; Wafa, L.A.; Cheng, H.; Zoubeidi, A.; Fazli, L.; Gleave, M.; Snoek, R.; Rennie, P.S. TAF1 Differentially Enhances Androgen Receptor Transcriptional Activity via Its N-Terminal Kinase and Ubiquitin-Activating and -Conjugating Domains. *Mol. Endocrinol.* **2010**, *24*, 696–708, doi:10.1210/me.2009-0229.

70. Dremel, S.E.; DeLuca, N.A. Genome Replication Affects Transcription Factor Binding Mediating the Cascade of Herpes Simplex Virus Transcription. *Proc. Natl. Acad. Sci.* **2019**, *116*, 3734–3739, doi:10.1073/pnas.1818463116.

71. Lester, J.T.; DeLuca, N.A. Herpes Simplex Virus 1 ICP4 Forms Complexes with TFIID and Mediator in Virus-Infected Cells. *J. Virol.* **2011**, *85*, 5733–5744, doi:10.1128/JVI.00385-11.

72. Gao, S.; Wang, F.; Sun, W.; Qian, X.; Ji, Y.; Cheng, Y.; Wang, X.; Liu, L.; Sheng, R.; Zeng, M. Preliminary Radiogenomic Study of Hepatitis B Virus-Related Hepatocellular Carcinoma: Associations between MRI

Features and Mutations. *Per. Med.* **2023**, doi:10.2217/pme-2022-0093.

73. Zhang, J.; Zhang, J.; Liu, W.; Ge, R.; Gao, T.; Tian, Q.; Mu, X.; Zhao, L.; Li, X. UBTF Facilitates Melanoma Progression via Modulating MEK1/2-ERK1/2 Signalling Pathways by Promoting GIT1 Transcription. *Cancer Cell Int.* **2021**, *21*, 543, doi:10.1186/s12935-021-02237-8.
74. Lum, K.K.; Howard, T.R.; Pan, C.; Cristea, I.M. Charge-Mediated Pyrin Oligomerization Nucleates Antiviral IFI16 Sensing of Herpesvirus DNA. *MBio* **2019**, *10*, doi:10.1128/mBio.01428-19.
75. Wang, X.; Li, J.; Cao, Y. Crosstalk between YY1 and LncRNAs in Cancer: A Review. *Medicine (Baltimore).* **2022**, *101*, e31990, doi:10.1097/MD.0000000000031990.
76. Broyles, S.S.; Liu, X.; Zhu, M.; Kremer, M. Transcription Factor YY1 Is a Vaccinia Virus Late Promoter Activator. *J. Biol. Chem.* **1999**, *274*, 35662–35667, doi:10.1074/jbc.274.50.35662.
77. Khachigian, L.M. The Yin and Yang of YY1 in Tumor Growth and Suppression. *Int. J. cancer* **2018**, *143*, 460–465, doi:10.1002/ijc.31255.
78. Knutson, B.A.; Oh, J.; Broyles, S.S. Downregulation of Vaccinia Virus Intermediate and Late Promoters by Host Transcription Factor YY1. *J. Gen. Virol.* **2009**, *90*, 1592–1599, doi:10.1099/vir.0.006924-0.
